# Genetic characterisation of the Connemara pony and the Warmblood horse using a within-breed clustering approach

**DOI:** 10.1186/s12711-023-00827-w

**Published:** 2023-08-17

**Authors:** Victoria Lindsay-McGee, Enrique Sanchez-Molano, Georgios Banos, Emily L. Clark, Richard J. Piercy, Androniki Psifidi

**Affiliations:** 1 Royal Veterinary College, London, UK; 2 The Roslin Institute, University of Edinburgh, Edinburgh, UK; 3 Scotland’s Rural College (SRUC), Edinburgh, UK; 4 Present Address: Royal (Dick) School of Veterinary Studies, University of Edinburgh, Edinburgh, UK

## Abstract

**Background:**

The Connemara pony (CP) is an Irish breed that has experienced varied selection by breeders over the last fifty years, with objectives ranging from the traditional hardy pony to an agile athlete. We compared these ponies with well-studied Warmblood (WB) horses, which are also selectively bred for athletic performance but with a much larger census population. Using genome-wide single nucleotide polymorphism (SNP) and whole-genome sequencing data from 116 WB (94 UK WB and 22 European WB) and 36 CP (33 UK CP and 3 US CP), we studied the genomic diversity, inbreeding and population structure of these breeds.

**Results:**

The k-means clustering approach divided both the CP and WB populations into four genetic groups, among which the CP genetic group 1 (C1) associated with non-registered CP, C4 with US CP, WB genetic group 1 (W1) with Holsteiners, and W3 with Anglo European and British WB. Maximum and mean linkage disequilibrium (LD) varied significantly between the two breeds (mean from 0.077 to 0.130 for CP and from 0.016 to 0.370 for WB), but the rate of LD decay was generally slower in CP than WB. The LD block size distribution peaked at 225 kb for all genetic groups, with most of the LD blocks not exceeding 1 Mb. The top 0.5% harmonic mean pairwise fixation index (F_ST_) values identified ontology terms related to cancer risk when the four CP genetic groups were compared. The four CP genetic groups were less inbred than the WB genetic groups, but C2, C3 and C4 had a lower proportion of shorter runs of homozygosity (ROH) (74 to 76% < 4 Mb) than the four WB genetic groups (80 to 85% < 4 Mb), indicating more recent inbreeding. The CP and WB genetic groups had a similar ratio of effective number of breeders (N_eb_) to effective population size (N_e_).

**Conclusions:**

Distinct genetic groups of individuals were revealed within each breed, and in WB these genetic groups reflected population substructure better than studbook or country of origin. Ontology terms associated with immune and inflammatory responses were identified from the signatures of selection between CP genetic groups, and while CP were less inbred than WB, the evidence pointed to a greater degree of recent inbreeding. The ratio of N_eb_ to N_e_ was similar in CP and WB, indicating the influence of popular sires is similar in CP and WB.

**Supplementary Information:**

The online version contains supplementary material available at 10.1186/s12711-023-00827-w.

## Background

When maintaining healthy animal populations, it is vital to sustain genetic variation. To achieve this, controlling the rate of inbreeding and preserving effective population size (N_e_) are essential, and can not only limit the loss of genetic variation but can prevent inbreeding depression affecting animal health and fertility [1].

Currently, there are over 350 distinct horse breeds ranging from the Shetland pony and the Clydesdale to the Arabian and the Thoroughbred [2]. Due to artificial selection for different performance, gait, resilience and colour traits, these breeds are genetically distinct from one another and, in the case of the less common breeds, they often have limited genetic diversity [3]. While many breeds are no longer exposed to the harsh environmental conditions to which they originally adapted to survive, a reduced population size decreases the genetic diversity, thus reduces future ability to adapt [4]. A reduced population size also increases the accumulation of deleterious alleles, thus increases the frequency of animal health problems and leads to reduction in fitness. Therefore, the study and subsequent management of these different horse breeds are important, including the monitoring of effective population size and levels of inbreeding.

The Connemara pony (CP) is an Irish native pony breed that is popular worldwide, but particularly in Ireland and the UK. CP were originally used for agriculture, including transportation of heavy weights across rough landscape, which led to a hardy native pony type. Breeds such as the Arabian, Shire, Thoroughbred, Welsh Cob, Hackney, Andalusian and Irish Draught all contributed to the formation of the early CP breed [5]. The Connemara Pony Breeders’ Society was established in 1923 [6, 7] and the first volume of the studbook was published in 1926, based on the selection of five stallions and 126 mares as initial breeding stock. The studbook ‘closed’ to outside blood in 1964, meaning that all registered ponies after this date must have both parents registered. CP are now so popular that there are 17 international daughter breed societies [8].

Since the 1970s, the aims of the CP Breeders’ Society (CPBS) shifted from breeding a traditional working pony, with the associated hardiness and bone width, to breeding a sports pony [7, 8] “of necessity lighter in bone and general structure” [9]. CP and CP crossbreeds are now common in athletic equestrian sports such as eventing and show jumping, with purebreds particularly common at the junior and Pony Club level. These new breeding goals diverge considerably from the breeding goals of those breeders who continue to breed for the traditional conformation for the show ring [5]. In the show ring, ponies are judged subjectively on their morphology and gaits against an agreed breed standard, rather than on their sporting performance. However, the aims of sport performance breeding have over time been incorporated into the show ring with the establishment of additional specific performance classes at the major breed shows during the 2000s [5].

The CP has a relatively small population size compared to many popular horse breeds: 108 stallions and 1204 mares were registered in Volume 24 of the CPBS Studbook in 2012 [5], and the smaller daughter studbook of the British Connemara Pony Society (BCPS) in 2019 [10] contained seven British-bred and 14 internationally-bred stallions, 77 British-bred and 36 internationally-bred mares and 91 British-born 2019 foals (including those British-born foals of Irish CPBS-registered parents). However, the CP breed does not suffer from the extremely small population sizes of the majority of UK native pony breeds [11], of which all but the Shetland pony are considered rare to endangered. The comparatively larger population size is possibly due to the CP’s unique popularity as modern sports ponies.

However, in spite of its popularity, there is at least one known autosomal recessive disease specific to the CP breed that is regularly tested for as part of the registration process, i.e. hoof wall separation disease (HWSD) [12]. The carrier frequency of HWSD was estimated at 14.8% [12], and concerns on the potential loss of genetic diversity in the breed by excluding carriers from breeding have led to official advice from the CBPS [13] and BCPS [14] not to exclude carriers from the gene pool, and rather to avoid breeding two carriers together to reduce risk of HWSD-affected offspring. This indicates concern that perhaps the effective population size is far smaller than the census population and the breed’s overall popularity suggest, and that an action to preserve its genetic diversity may be required.

CP have previously been compared to other UK native pony breeds [15–18] using population structure methods such as multidimensional scaling, hierarchical clustering, and the Bayesian STRUCTURE algorithm [19] on short sequence repeats, single nucleotide polymorphism (SNP) data and mitochondrial DNA sequences. CP consistently appear to be closely related to Highland and Welsh ponies, and also to the Irish Draught and, therefore, to the Irish Sports Horse [17, 20].

Another horse breed that is popular in equestrian sports similar to those of the CP is the Warmblood horse (WB). The WB is a middleweight horse type that has been selectively bred in various European countries for light farm work and cavalry use since the eighteenth century [21, 22]. Since the Second World War, the WB is no longer used for these purposes but instead is very popular for sports, particularly dressage and show jumping for which they are now selectively bred [23]. Indeed, the genetic contribution to this type of sporting performance has been well studied [22, 24–29]. The number of WB is much larger than that of CP but, in Germany, the number of WB foals being produced is decreasing. Germany is the largest producer of WB with approximately 39,000 foals per year across its studbooks during the 1990s [21], but only 25,560 and 27,615 foals in 2018 [30] and in 2022 [31, 32], respectively. In the UK, approximately 12.4 to 14% of all horses are WB [33–35].

Unlike the CP, the WB is not a closed population breed and is traditionally defined by the country or region from which the horse originates, forming regional subpopulations [21]. While there are many different European Warmblood studbooks that register WB horses, with some countries such as Germany having many, the only closed Warmblood studbook is the Trakehner Studbook. Across other WB studbooks, many stallions are approved for offspring registration in multiple different studbooks, and offspring can be registered in a different studbook than their sire and/or dam. Previous studies using genomic data have struggled to differentiate the WB subpopulations registered with these different studbooks (aside from the Trakehner) due to the levels of admixture between studbooks [29, 36].

Petersen et al. [3] compared the WB breed to many other horse breeds including Thoroughbreds, Arabians, Iberian, draft and pony breeds. While they did not compare WB to CP, it was clear from the expected heterozygosity, parsimony and principal component analyses that the WB is genetically distinct from the UK native pony breeds and draught breeds. This likely indicates that WB are very distinct from CP although the current breeding goals for both breeds are similar.

Several parameters based on genetic data measure the genetic diversity of a population. Effective population size (N_e_), which is an idealised population size that undergoes genetic drift at the rate of the real-life population and was first described by Wright in 1931 [37], captures the degree of inbreeding and overall genetic variation in populations for which the census population size may not. N_e_ can be calculated in a variety of ways, including based on linkage disequilibrium (LD) as r^2^ using genome-wide genotype data [38], which reflects not only the recombination rate between different loci but also the degree of admixture and effect of genetic drift. Closely related is the effective number of breeders (N_eb_), which describes the number of breeding adults in the previous generation. N_eb_ is nearly equal to N_e_ in populations in which the generations do not overlap and the population consists of reproductive adults [39]. One method to calculate N_eb_ is the molecular coancestry method, based on alleles that are identical-by-state between individuals [40]. Another important genetic metric is inbreeding, due to the parents sharing one or more ancestors, which can lead to loss of genetic diversity when inbreeding levels are high at the population level. While inbreeding can be calculated from pedigree data, inbreeding coefficients calculated from genetic data are often considered more accurate [41–43]. One method of calculating inbreeding from genetic data considers the runs of homozygosity (ROH), based on the fact that increased homozygosity due to inbreeding is usually inherited in tracts, with a random distribution across the genome compared to a specific pattern of homozygosity in outbred individuals due to the recombination rate in specific genomic regions [44]. All of these measures are useful metrics of the genetic diversity of populations based on genetic data, which provide further information than pedigree-based studies alone, for evidence-based management of breeds.

In the present study, we assessed the genomic characteristics and genetic variability in the athletic CP and WB breeds. Molecular estimates of co-ancestry and inbreeding using ROH were compared between the two breeds as well as within breed subpopulations, to further characterise them and better understand the impact of selection practices in these breeds.

## Methods

### Dataset

Genetic data from the UK-based Connemara ponies (n = 34) and Warmblood horses (n = 97) used in this project were collected for another study using a combination of random sampling, voluntary response sampling and snowball sampling. Briefly, we had access to muscle biopsy samples from 62 horses (16 CP and 46 WB), and blood samples from six horses (2 CP and 4 WB). Sixty-three other horses (16 CP and 47 WB) were recruited via the Royal Veterinary College website, social media and stakeholder groups, from across the UK and a range of different sporting disciplines, with a hair root sample provided for each one. Thirty four CP represents just over 1/3 of the number of foals registered with the BCPS in 2019 [10]. The mean age of all horses was 10.64 (ranging from 2 to 26 years; sd = 4.32) years old and 61.18% of the samples were males and 37.50% were females (sex was not recorded in a small number of cases).

### DNA extraction, genotyping and sequencing

DNA was extracted using the following three methods: for muscle tissue, the Qiagen DNEasy Blood and Tissue kit was used according to manufacturer’s instructions; for whole blood, the Illustra Nucleon BACC kit was used according to manufacturer’s instructions; for hair root, the Qiagen Gentra Puregene kit was used according to manufacturer’s instructions (see Additional file 1: Methods S1). Of these, 17 CP and 79 WB were genotyped using the Affymetrix 670k HD Equine SNP array [45], and 19 CP and 19 WB were whole-genome sequenced (WGS) at 15X coverage using the Illumina HiSeqX 150 bp paired-end sequencing technology, with three individuals that were both genotyped and sequenced. In addition, non-UK WGS data from 22 European WB and four US CP were downloaded from publicly available sources (NCBI SRA BioProjects PRJEB14779 for WB and PRJNA273402 for CP) and combined with the UK samples previously described (sample details are in Additional file 2: Table S1). Prior to merging, all sequencing reads were mapped to EquCab3.0 and variants were called using the GATK4 Best Practices pipeline [46, 47]. Only the biallelic SNPs that overlapped with the Affymetrix array were kept and, after filtering, the data were merged with the UK-based genotypes. Then, the merged dataset underwent the following quality control thresholds using the PLINK 1.9 software [48]: a 95% call rate per sample and per SNP, a 1% minor allele frequency (MAF), and a *p* value for the Hardy–Weinberg equilibrium test > 10e^−6^. After quality control, 152 samples (36 CP and 116 WB) and 446,878 SNPs remained for further analysis, referred to hereafter as genotype data.

Metadata for each sample included their breed subtype, based on the relevant registered studbook (Table 1) and the origin. Not all horses had pedigree data available, so pedigree measures of inbreeding were not calculated. Comparative analyses were performed between different sample groupings: (1) the horse breed (CP or WB); (2) the breed subtype (based on registered studbook, Table 1); (3) origin (UK, rest of Europe [abbreviated to EU WB] or US); and (4) the within-breed genetic group, as identified by k-means clustering, as discussed below.

**Table 1:** Breed subtype groupings of sample horses

Breed	Breed subtype group	Breed studbooks included	Country of studbook	Number of samples
CP	CP	Connemara	Ireland	31
	CP X	Connemara (non-registered)	Ireland	5
WB	Trakehner		Germany	5
	Hanoverian		Germany	6
	Holsteiner		Germany	9
	Oldenburg		Germany	6
	Westphalian		Germany	3
	Zangersheide		Germany	3
	Belgian WB		Belgium	6
	Dutch WB	KWPN studbook	Netherlands	18
	Selle Français		France	3
	Anglo European		UK	9
	British WB		UK	4
	Other WB	Polish WB	Poland	1
		Slovakian WB	Slovakia	1
		Performance Sales International (PSI)	Germany	1
		Baden-Württemberger	Germany	1
		Bavarian WB	Germany	1
		Swiss WB	Switzerland	2
		Trakehner x WB	–	1
		KWPN x Selle Français	–	1
	WB X	Dutch WB (non-registered)	–	1
		Polish WB (non-registered)	–	1
		Unknown WB (non-registered)	–	2
	WB	Unknown WB	–	31

*CP* Connemara pony, *WB* Warmblood horse, *KWPN* Koninklijk Warmbloed Paardenstamboek Nederland, *PSI* Performance Sales International, *UK* United Kingdom, *WB X* non-registered Warmbloods

### Principal component analysis

Genomic relationship matrices (GRM) were computed both within and across breeds, and decomposed through principal component analysis (PCA) that was performed using the GEMMA algorithm [49]. Principal components (PC) were then plotted in Python 3.7 using the seaborn [50] and matplotlib [51] packages. Kernel density estimator (KDE) plots, a non-parametric method of smoothing a density estimation [50] analogous to a histogram, were also produced for each group for each PC.

K-means clustering based on the PCA was used to identify any within-breed genetic groups. Elbow plots (using total within sum of squares method) and silhouette plots were produced using the R package factoextra [52] to determine the optimal number of genetically distinct groups. Association of breed subtypes and sample origin location to these distinctive groups was performed using Chi-square tests in the Python 3 statsmodels package [53].

### Linkage disequilibrium analysis

The genotype data were split according to the within-breed genetic groups identified with k-means clustering, and SNPs were thinned to 20 SNPs per Mb using the mapthin (v1.11) program [54], resulting in 46,606 SNPs per breed per dataset. Linkage disequilibrium (LD) was computed as pairwise r^2^ using the PLINK 1.9 software, with the maximum window size being equal to the largest equine chromosome (*Equus caballus* chromosome (ECA)1, i.e. 188.26 Mb in EquCab3.0). LD decay was plotted using the R packages dplyr [55], stringr [56] and ggplot2 [57], and maximum block size for subsequent LD block analysis was derived from the minimum distance at which LD reached the mean. LD blocks were then computed using PLINK 1.9 and plotted in R using the above packages—however, due to their small sample size, estimates were not calculated for the genetic groups C2, C4, W1 and W3. LD decay and block analyses were performed for each breed (CP and WB), and for each within-breed genetic group.

### Effective population size

Historical effective population size (N_e_) was calculated based on the full genotyping data from the autosomes, which were split according to within-breed genetic groups, from 13 to 999 prior generations using the LD-based method of the SNeP program [38], and the Sved and Feldman [58] recombination rate modifier, with the following equation [59]:

$$N_{t} = \left( {4f\left( {c_{t} } \right)} \right)^{{ - 1}} (E[r_{{adj}}^{2} |c_{t} ]^{{ - 1}} - \alpha ),$$

where ${N}_{t}$ is the N_e_ at $t$ prior generations, ${c}_{t}$ is the recombination rate for a specific physical distance between loci (assuming 1 cM ≈ 1 Mb), ${r}_{adj}^{2}$ is the LD adjusted for sample size and $\alpha$ is a correction for the occurrence of mutations. Ordinary least squares regression using the LinearRegression command from the scikit-learn package [60] in Python 3.7 was used to calculate the N_e_ at the current generation ($t$ = 0,the y-intercept) for each within-breed genetic group.

The thinned PLINK files were recoded to GENEPOP format for the autosomes only using PGDSpider [61], in order to estimate the effective number of breeders (N_eb_) using NeEstimator [62] with the molecular co-ancestry (MCoA) method [40]:

$${\widehat{N}}_{eb}=\frac{1}{2\widehat{{f}_{1}}},$$

where $\widehat{{f}_{1}}=\frac{1}{{n}_{p}}\sum_{x=1}^{n}\sum_{y>x}^{n}{\widehat{f}}_{1,xy},$ with ${n}_{p}$ as $n(n-1)/2$ pairs, and ${\widehat{f}}_{1,xy}$ is the average parent-based ancestry between individuals $x$ and $y$, calculated as:

$${\widehat{f}}_{1,xy}=\frac{1}{\sum_{l=1}^{L}{w}_{l}}\sum_{l=1}^{L}{w}_{l}\frac{{f}_{M,xy,l}-{\widehat{s}}_{l}}{1-{\widehat{s}}_{l}},$$

and ${w}_{l}=\frac{{(1-{\widehat{s}}_{l})}^{2}}{\sum_{i=1}^{{n}_{l}}{{\widehat{p}}_{i}}^{2}(1-\sum_{i=1}^{{n}_{l}}{{\widehat{p}}_{i}}^{2})},$ where ${\widehat{p}}_{i}$ is the estimated frequency of allele $i$ at locus $l$ across samples, ${s}_{l}$ represents the probability of two alleles at locus $l$ being identical-by-state, $L$ is the number of loci, and ${f}_{M,xy,l}$ is the molecular similarity index between individuals $x$ and $y$ at locus $l$. Estimates of N_eb_ were calculated for each within-breed genetic group.

### Estimates of genetic diversity and signatures of selection using the fixation index (FST)

Metrics for genetic diversity and hierarchical F-statistics were calculated using the hierfstat package in R [63] on the non-thinned data. Mean alternate allelic frequency, observed heterozygosity (H_O_), within-population gene diversity (H_S_), and Wright’s F-statistics, including fixation index (F_ST_) and individual inbreeding coefficient by expected heterozygosity (F_IS_), were calculated. Overall F_ST_ was calculated hierarchically for within-breed genetic groups and within-breed breed types in the total population.

Furthermore, F_ST_ was calculated per marker between all CP and all WB samples using PLINK 1.9 [48]. Then, pairwise F_ST_ values per marker were calculated using PLINK 2 [64] for each pairwise analysis between genetic groups. In order to compare across multiple genetic groups, the harmonic mean F_ST_ was also calculated from the pairwise comparisons using the Scipy package [65] in Python 3.7 for each marker. Ten comparisons were performed using harmonic mean F_ST_ pairwise estimates: between all pairwise CP within-breed genetic group comparisons; between all pairwise WB within-breed genetic group comparisons; and between the three within-breed comparisons for each of the eight genetic groups individually. Results were plotted using the R package qqman [66]. The SNPs with the top 0.5% of F_ST_ values or from all SNPs with an F_ST_ > 0.1 (the threshold producing the smallest number of SNPs in each instance) from each of the 11 comparisons were identified.

Genes within 1 Mb of the top 0.5% of markers from the breed comparison and the two harmonic mean comparisons were extracted using the BiomaRt package in R [67, 68]. The identified genes were then assessed using an over-representation test in the Database for Annotation, Visualization and Integrated Discovery (DAVID) [69] for significant curated database terms to indicate particular overrepresented pathways or processes that are subject to selection [70–74]. DAVID is a publicly available tool for gene enrichment analysis, which provides functional analysis of large gene lists by mapping a list of genes of interest to the relevant annotation (e.g. Gene Ontology (GO) terms [70, 74]) and using statistical testing to highlight enriched or overrepresented GO terms. The settings used were the official gene symbols, the *Equus caballus* background, an EASE threshold of 0.1, and a Benjamini-Hochberg-corrected p-value of 0.05.

### Runs of homozygosity

Runs of homozygosity (ROH) were detected for each individual sample on the autosomes using the detectRUNS package in R [75] on the non-thinned data. The settings for ROH detection were made equivalent to PLINK defaults, except for minimum ROH length (derived from our LD analyses), minimum density (1 SNP per 60 kb) and maximal gap (500 kb) which were derived from Meyermans et al. [76], where the effects of various ROH detection parameters on animal genotyping data were examined. ROH present in at least 10% of individuals [77] (with a minimum of 2) of specific groups (within-breed genetic clusters, origin, or breed), were identified and selected using the bedtools multiinter tool [78, 79]. These selected ‘common’ ROH were also compared to identify those that were shared by multiple groups. Genes within all of these ROH were identified using the Ensembl BioMart tool [80], and assessed using an over-representation test in DAVID [69] with the official gene symbols, the *Equus caballus* background, an EASE threshold of 0.1, and a Benjamini-Hochberg-corrected p-value of 0.05.

### Genomic inbreeding

Genomic inbreeding was then calculated based on the extent of ROH for each individual as follows [44]:

$${F}_{ROH}=\frac{\sum {ROH}_{length}}{{Length}_{genome}},$$

where $\sum {ROH}_{length}$ is the total length of identified ROH in a given individual, and ${Length}_{genome}$ is the total length of the equine autosomes. ${F}_{ROH}$ was calculated at both the chromosome-wide and genome-wide levels and compared between origin groups as well as between within-breed genetic groups. ${F}_{ROH}$ was then compared using one-way ANOVA (to compare within-breed genetic groups, and separately origingroups) to identify differences in inbreeding. ROH were also split into classes ranging from 1 to 2 Mb, 2 to 4 Mb, 4 to 8 Mb, 8 to 16 Mb and more than16 Mb to assess recent versus ancient inbreeding [81].

## Results

### Principal components analysis

**Fig. 1:**
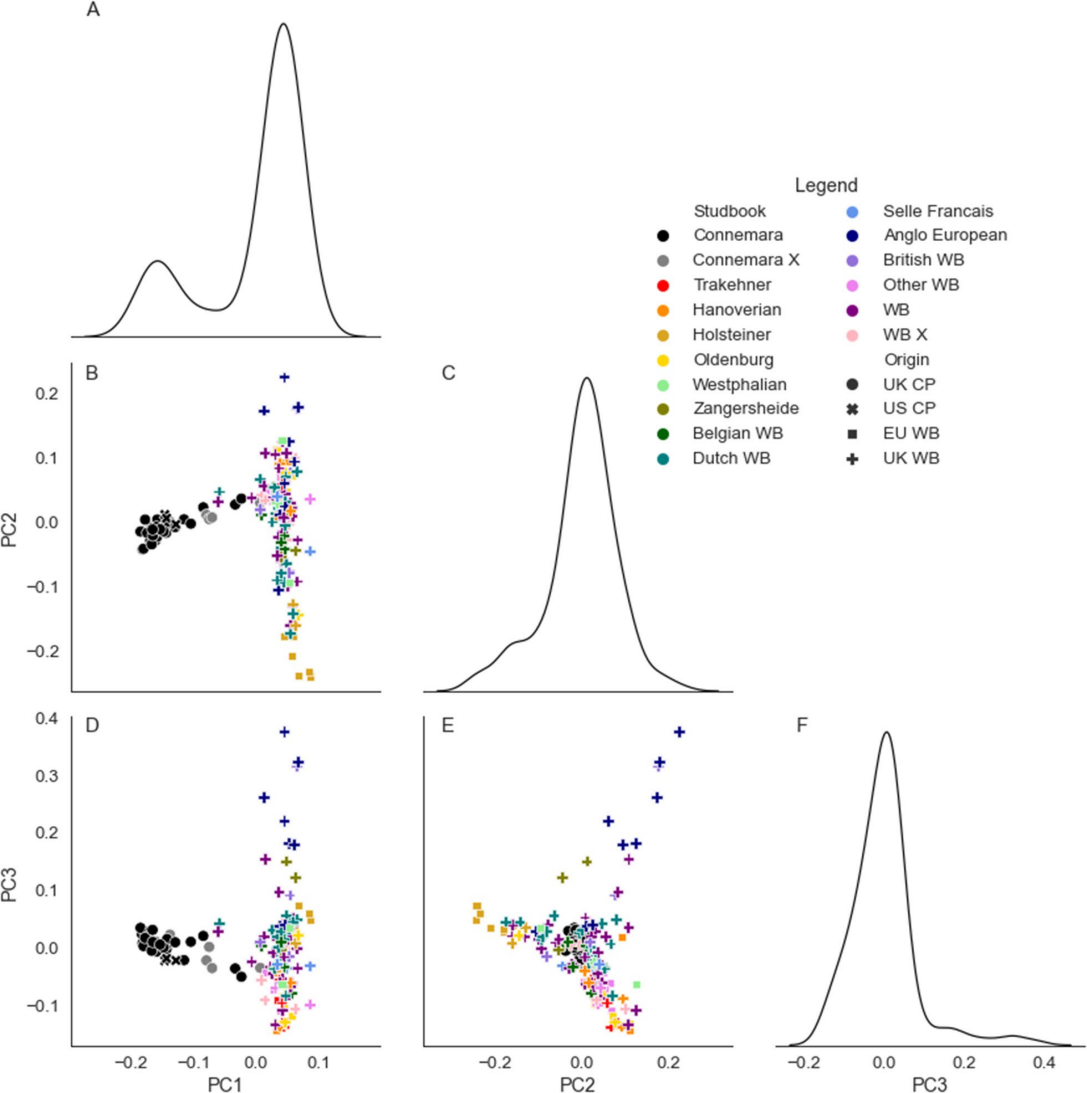
Principal components (PC) of the genetic relationship matrix for 116 WB and 36 CP. The three lower diagonal plots show principal components analysis (PCA) biplots, with colour designating the breed subtype and marker designating the sample origin: **B** principal component (PC) 1 by PC 2; **D** PC 1 by PC 3; and **E** PC 2 by PC 3. Diagonal plots are kernel density estimator plots illustrating the distributions of the principal components: **A** of PC 1; **C** of PC 2; and **F** of PC 3. The first three PCs explained 4.1%, 1.8% and 1.6% of variance respectively. *CP* Connemara pony, *WB* Warmblood horse, *UK* United Kingdom, *EU* rest of Europe, *US* United States, *X* unregistered

**Fig. 2:**
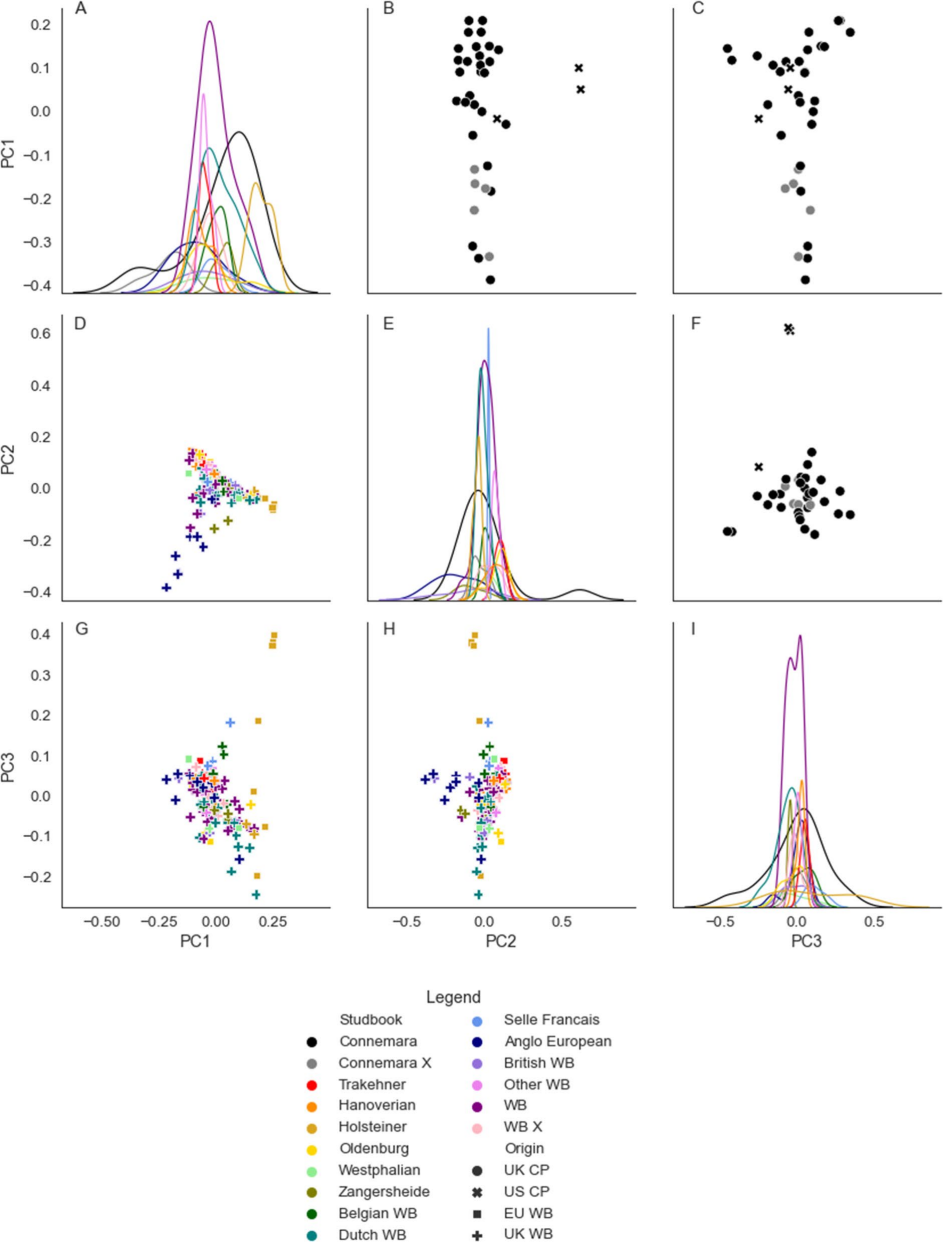
Principal components (PC) of the genetic relationship matrices for 116 WB (lower diagonal) and 36 CP (upper diagonal). Upper and lower diagonal plots show principal components analysis (PCA) biplots for CP and WB respectively, with colour designating the breed subtype and marker designating the sample origin. In CP: **B** principal component (PC) 1 by PC 2; **C** of PC 1 by PC 3; and **F** of PC 2 by PC 3; and in WB: **D** PC 1 by PC 2; **G** PC 1 by PC 3; and **H** PC 2 by PC 3. Diagonal plots are kernel density estimator plots illustrating the distributions of the principal components, with distribution curves for each breed subtype: distribution for both breed analyses is shown in: **A** PC 1; **E** PC 2; and **I** PC 3. The first three PC in CP explained 4.7%, 4.2% and 3.7% of variance respectively, and in WB explained 2.5%, 2.2% and 1.7% of variance respectively. *CP* Connemara pony, *WB* Warmblood horse, *UK* United Kingdom, *EU* rest of Europe, *US* United States, *X* unregistered

CP and WB separated along PC1, with only some WB individuals that include Irish Sports Horses in their pedigree overlapping with CP (Fig. 1). There was evidence of separation along PC2 and PC3 of the Anglo European, British WB and Holsteiners. Other WB subtypes did not show genetic differentiation. Within-breed biplots of the PC and kernel density estimator (KDE) plots of the distribution across PC are presented in Fig. 2. The separation of non-registered CP (CP X) became apparent, as well as the separation of the Anglo European and British WB and the Holsteiners (as in Fig. 1). Clustering analyses suggested that the appropriate number of distinct genetic groups within each breed was 4 (see Additional file 3: Fig. S1). Animals were then assigned to these within-breed genetic groups using the k-means method (see Additional file 4: Fig. S2).

Genetic groups identified in the k-means analyses were compared with the breed subtypes using Chi-square tests (to assess overrepresentation of particular subtypes in certain within-breed genetic groups) (see Additional file 5: Table S2). In addition, following the results of the PCA analyses, the origin of the samples (UK vs. US in CP and UK vs. EU in WB) was compared to the available breed subtypes (see Additional file 5: Table S2). Only Holsteiner, Anglo European and British WB were associated with a particular genetic group (see Additional file 5: Table S2).

### Linkage disequilibrium analysis

**Fig. 3:**
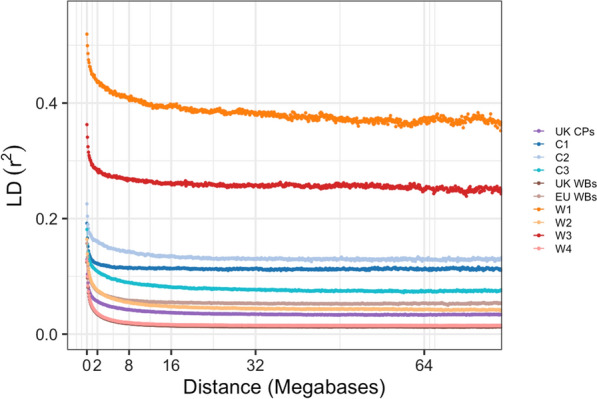
Linkage disequilibrium (LD; pairwise r^2^) decay plot for CP and WB within-breed genetic groups and sample origin groups. *CP* Connemara pony, *WB* Warmblood horse; *UK* United Kingdom, *EU* rest of Europe

CP genetic group C4 and US CP were excluded from this analysis due to their small group sample sizes (for C4 n = 2 and for US CP n = 3). LD decayed exponentially in both CP and WB, with the maximum r^2^ ranging from 0.124 to 0.187 depending on the origins (Fig. 3). LD decay had a low range of mean LD (0.013 to 0.054), with CP having the middle value between WB origin groups, a trend that was also observed in the maximum and minimum LD values. However, CP had lower LD decay than WB both for window sizes between 0 and 1 Mb and between 2 and 4 Mb.

When comparing different genetic groups, the maximum LD varied greatly, ranging from 0.122 to 0.258 in the CP genetic groups and from 0.127 to 0.519 in the WB genetic groups. Mean r^2^ also varied considerably, ranging from 0.077 to 0.130 in the CP within-breed genetic groups and from 0.016 to 0.370 in the WB within-breed genetic groups.

**Fig. 4:**
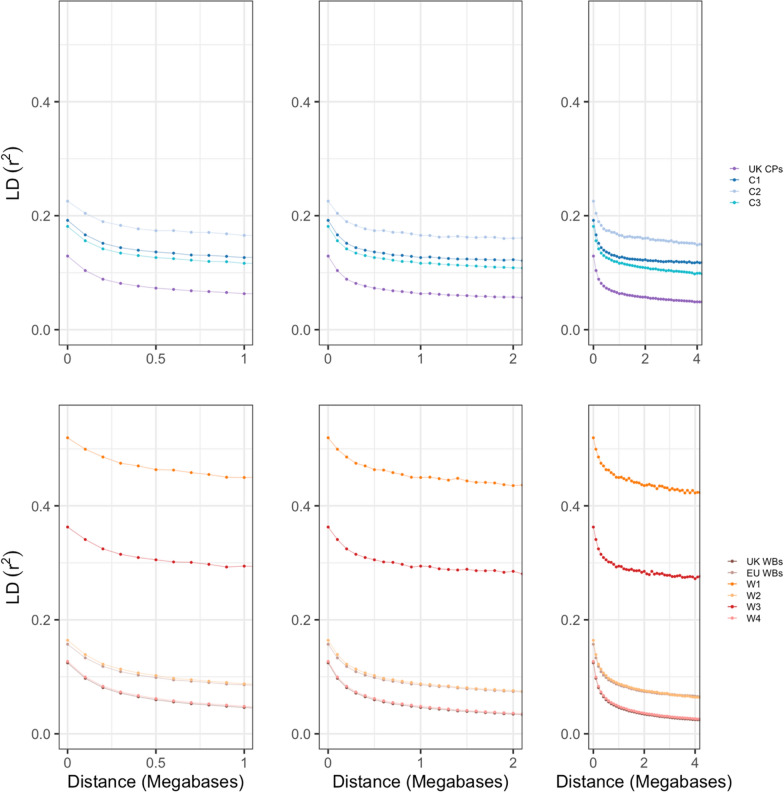
Linkage disequilibrium (pairwise r^2^) decay plot. Linkage disequilibrium (LD) decay plot for **A** CP within-breed genetic groups and sample origin groups between 0 and 1 Mb; **B** CP within-breed genetic groups and sample origin groups between 0 and 2 Mb; **C** CP within-breed genetic groups and sample origin groups between 0 and 4 Mb; **D** WB within-breed genetic groups and sample origin groups between 0 and 1 Mb; **E** WB within-breed genetic groups and sample origin groups between 0 and 2 Mb; and **F** WB within-breed genetic groups and sample origin groups between 0 and 4 Mb. *CP* Connemara pony, *WB* Warmblood horse, *UK* United Kingdom, *EU* rest of Europe

In general, LD in CP within-breed genetic groups showed greater values and a slower decay than in WB within-breed genetic groups (Fig. 4 and Table 2), with a slower rate of decay, which is particularly noticeable under 2 Mb (Fig. 4). Rate of decay between 0 and 1 Mb was also significantly lower in CP within-breed genetic groups than in WB within-breed genetic groups (independent samples t-test, p = 0.005), as well as those between 1 and 2 Mb (p = 0.0004) and between 2 and 4 Mb (p = 0.03).

**Table 2:** Comparison of linkage disequilibrium (r^2^) between CP and WB within-breed genetic groups and sample origin groups

Genetic group/Sample origin	Mean	Max	Min	Rate of decay 0–1 Mb (r^2^/Mb)	Rate of decay 1–2 Mb (r^2^/Mb)	Rate of decay 2–4 Mb (r^2^/Mb)
UK CP	0.035	0.129	0.010	0.066	0.006	0.004
C1	0.113	0.192	0.001	0.065	0.004	0.003
C2	0.130	0.258	0.013	0.060	0.006	0.003
C3	0.077	0.181	0.023	0.065	0.007	0.005
UK WB	0.013	0.124	0.000	0.078	0.012	0.005
EU WB	0.054	0.187	0.021	0.071	0.012	0.004
W1	0.370	0.519	0.111	0.069	0.015	0.006
W2	0.043	0.164	0.012	0.076	0.012	0.006
W3	0.257	0.363	0.018	0.069	0.009	0.007
W4	0.016	0.127	0.004	0.079	0.012	0.005

*LD* linkage disequilibrium, *CP* Connemara pony, *WB* Warmblood horse, *UK* United Kingdom, *EU* Rest of Europe

**Fig. 5:**
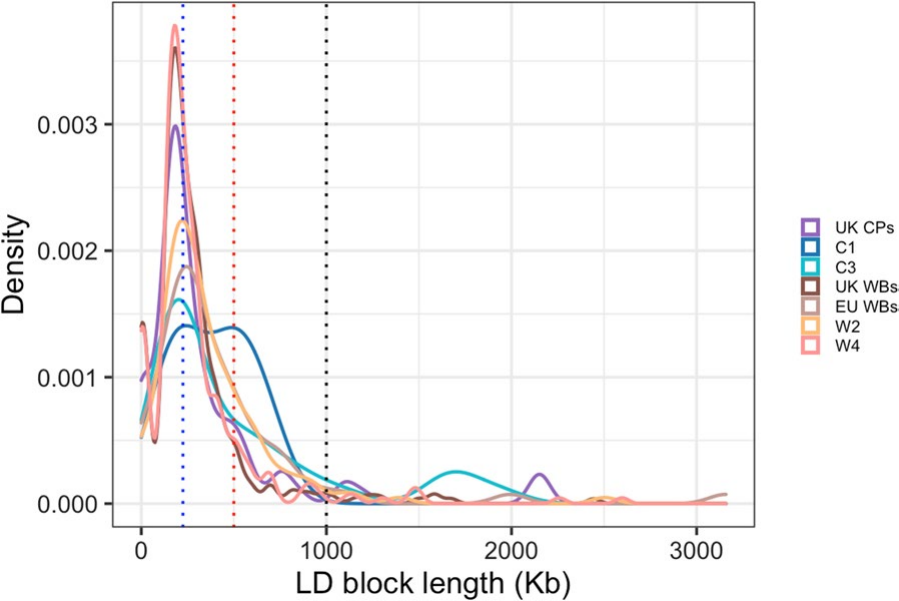
LD block density by length. Linkage disequilibrium (LD) block density by length in kb in four within-breed genetic groups (C1 and C3, and W2 and W4) and three sample origin locations (UK CP, UK WB and EU WB). Peak density for all groups was at approximately 225 kb (blue vertical line), except for C1 which had a second peak at approximately 500 kb (red vertical line). 1 Mb (black vertical line) captured the majority of LD blocks across genetic groups and origin locations. *CP* Connemara pony, *WB* Warmblood horse, *UK* United Kingdom, *EU* rest of Europe

As LD was close to the baseline in all groups by a 8-Mb window size (Fig. 3), this distance was used as maximum window size for the LD block analysis (Fig. 5). All groups presented blocks with left-skewed size distributions peaking at 225 kb (blue vertical line, Fig. 5). Most of these LD blocks were smaller than 1 Mb (black vertical line, Fig. 5). Notably, one genetic group (C1) presented a distribution of LD blocks peaking above 225 kb (Fig. 5). This genetic group was mainly associated with the non-registered CP, and the size distribution presented a second peak at 500 kb (red vertical line, Fig. 5). This second peak could indicate outbreeding in the non-registered CP when compared to registered CP: for the latter, both parents must be registered CP.

### Effective population size

With the variation in sample size between within-breed genetic groups and origin groups, comparison of N_e_ across all groups proved difficult. Table 3 illustrates the historical N_e_ intercept (N_eH_) and molecular co-ancestry estimates (MCoA N_eb_) for the four within-breed genetic groups with similar sample sizes (C1, C2, C3 and W3).

**Table 3:** Estimates of effective population size in selected CP and WB genetic groups

	Molecular co-ancestry		Historical N_e_ intercept (N_eH_)	Group sample size	$\frac{{N}_{eb}}{{N}_{eH}}$
	N_eb_	CI			
C1	8.6	8.3–8.9	67.8	10	0.13
C2	6.1	5.9–6.3	40.5	9	0.15
C3	5.7	5.5–5.9	78.3	15	0.07
W3	6.0	5.9–6.2	45.6	11	0.13

*CP* Connemara pony, *WB* Warmblood horse, *N*_*e*_ effective population size; *N*_*eb*_ effective number of breeders, *CI* confidence interval calculated using a jackknife approach [82]

In spite of a much larger N_eb_ and N_eH_ in C1, the genetic groups C1 and W3 had very similar ratios of N_eb_ to N_eH_. In contrast, C2 had the largest N_eb_, but the smallest N_eH_, while for C3 it was the opposite, with the largest N_eH_ and smallest N_eb_.

### Genetic diversity and fixation index (FST) analyses

Mean alternate allelic frequency, observed heterozygosity (H_O_), within-population expected heterozygosity (H_S_), and individual inbreeding coefficient by expected heterozygosity (F_IS_) were calculated per genetic group (Table 4). The two genetic groups with the lowest (C4) and highest (C1) mean alternate allele frequency, H_O_ and H_S_, were also the smallest group (C4) and the group with the highest level of expected admixture (significantly associated with non-registered CP) respectively. Notably, all genetic groups had higher H_S_ than H_O_, resulting in negative mean F_IS_ values—but both C4 and W1, which were the smallest sample size genetic groups with the lowest mean F_IS_, did not have an F_IS_ that significantly differed from 0. These results indicate a greater degree of genetic diversity within groups than expected, possibly due to the non-random mating in these horse breeds [83].

**Table 4:** Measures of genetic diversity per genetic group

Genetic group	Mean alternate allelic frequency (SD)	Mean H_O_ (SD)	Mean H_S_ (SD)	Mean F_IS_ (SD)
C1	0.1909 (0.1276)	0.3819 (0.2552)	0.3025 (0.1742)	*− 0.1978* (*0.1592*)
C2	0.1842 (0.1404)	0.3683 (0.2808)	0.2888 (0.1921)	*− 0.2045* (*0.1721*)
C3	0.1876 (0.1347)	0.3752 (0.2694)	0.2925 (0.1837)	*− 0.2100* (*0.1591*)
C4	0.1642 (0.1980)	0.3285 (0.3960)	0.2478 (0.2761)	− 0.3056 (0.4204)
W1	0.1801 (0.1823)	0.3602 (0.3646)	0.2643 (0.2416)	− 0.2978 (0.2736)
W2	0.1832 (0.1222)	0.3664 (0.2445)	0.2887 (0.1696)	− *0.2000* (*0.1408*)
W3	0.1826 (0.1424)	0.3651 (0.2849)	0.2819 (0.1936)	− *0.2192* (*0.1761*)
W4	0.1841 (0.1123)	0.3683 (0.2246)	0.2921 (0.1570)	− *0.1947* (*0.1272*)

F_IS_ values in italic indicate a p-value lower than 0.05 in a one-sample t-test, indicating that the F_IS_ is significantly different from 0

*SD* standard deviation, *H*_*O*_ observed heterozygosity, *H*_*S*_ within-population gene diversity, *F*_*IS*_ Wright’s inbreeding coefficient by expected heterozygosity

Differentiation was less pronounced between different studbooks than between either genetic groups or breed overall, particularly using weighted values (F_STP_ [84]; Table 5). When hierarchical F_ST_ was calculated for both the genetic group and studbook within breed, genetic group captured more genetic differentiation.

**Table 5:** F_ST_, F_STP_, and hierarchical F_ST_, between breeds, genetic groups and studbooks in CP and WB horses

	F_ST_	F_STP_	Hierarchical F_ST_ (genetic group within breed model)	Hierarchical F_ST_ (studbook within breed model)
Breed	0.0092	0.0182	0.0137	0.0157
Genetic group	0.0282	0.0321	0.0119	–
Studbook	0.0086	0.0092	–	0.0060

*F*_*ST*_ Wright’s fixation index, *F*_*STP*_ population-corrected F_ST_, *CP* Connemara pony, *WB* Warmblood horse

**Fig. 6:**
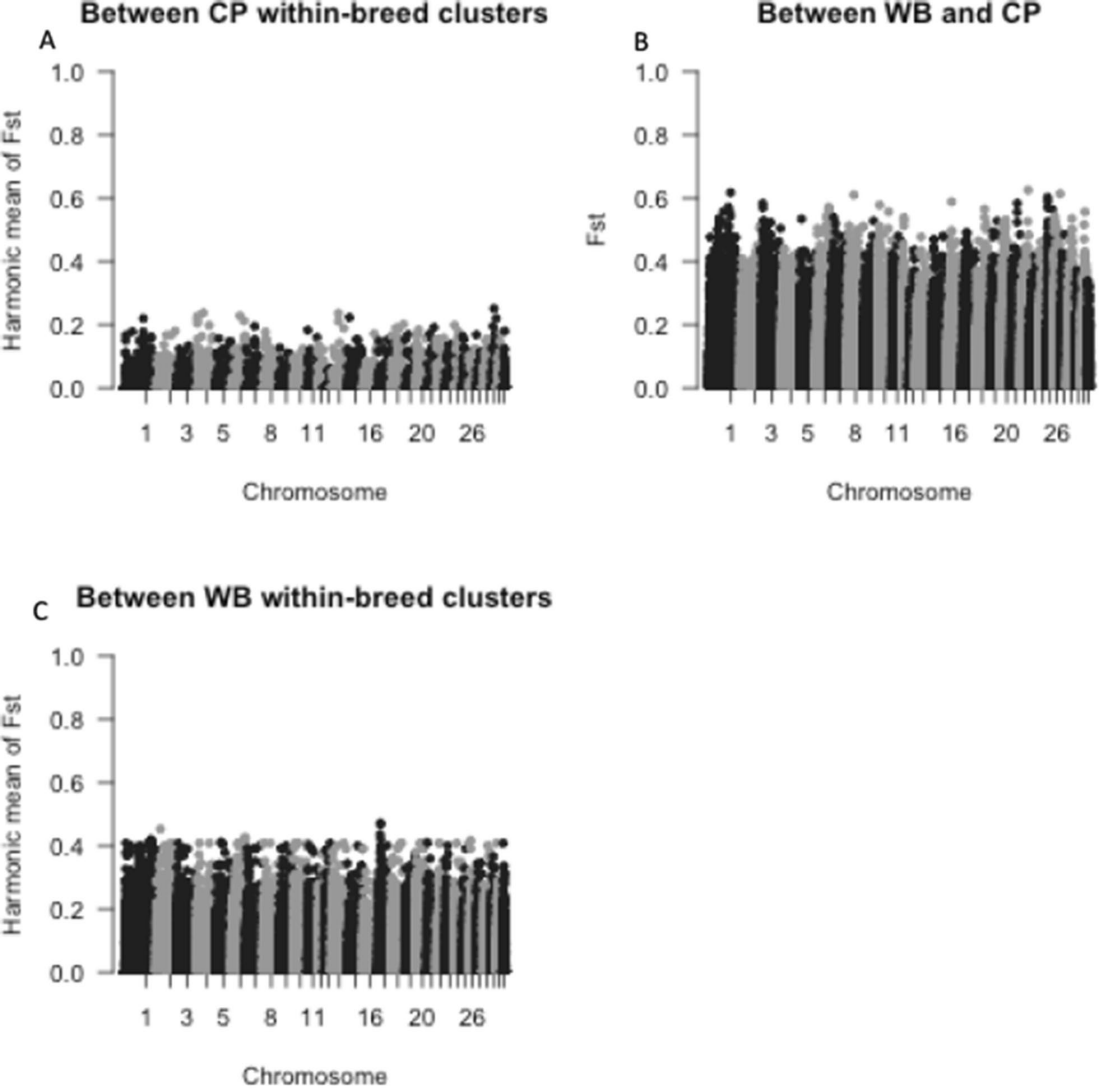
Manhattan plot of values. Manhattan plot of values between **A** all CP and WB, and the harmonic mean (HM) of the pairwise values between **B** all CP within-breed genetic groups, and between **C** all WB within-breed genetic groups (bottom left). *CP* Connemara pony, *WB* Warmblood horse, *F*_*ST*_ Wright’s fixation index

F_ST_ per marker was calculated between all CP and all WB, and pairwise F_ST_ values were calculated between genetic groups within each breed, per marker, with harmonic mean F_ST_ calculated within CP and within WB. Genetic groups C4 and W1 were excluded due to their small sample size. Results for all these comparisons are shown in Fig. 6. As expected, the differentiation between breeds (CP versus WB) is greater than the differentiation between genetic groups pertaining to the same breed.

The top 0.5% of F_ST_ values (2144 SNPs) ranged from 0.01 to 0.251 when comparing CP within-breed genetic groups, from 0.218 to 0.472 when comparing WB within-breed genetic groups, and from 0.334 to 0.626 when comparing the two breeds (Table 6). Notably, the harmonic mean F_ST_ within CP was the only group to have SNPs below F_ST_ = 0.1 in the top 0.5% of F_ST_ values. W3 also had the fewest genes located within 1 Mb of the top 0.5% F_ST_ SNPs of the genetic groups, indicating either a higher degree of overlap of high F_ST_ regions, or high F_ST_ in non-coding regions of the genome.

**Table 6:** Minimum and maximum F_ST_ for the top 0.5% of SNPs (or total number of SNPs where minimum F_ST_ is lower than 0.1) and total number of genes within 1 Mb of SNPs from within and across breed and genetic group fixation index analysis

Comparison	Minimum F_ST_ / number of SNPs with F_ST_ > 0.1	Maximum F_ST_	Number of genes within 1 Mb of the top 0.5% F_ST_ SNPs
Between CP and WB	0.334	0.626	6729
HM within CP	303	0.251	2522
HM within WB	0.218	0.472	7613
HM C1 against CP	0.266	0.701	7781
HM C2 against CP	0.227	0.542	4528
HM C3 against CP	0.195	0.505	6763
HM W2 against WB	0.170	0.446	5166
HM W3 against WB	0.314	0.725	333
HM W4 against WB	0.120	0.379	6755

*F*_*ST*_ Wright’s fixation index, *SNP* single nucleotide polymorphism, *CP* Connemara pony, *WB* Warmblood horse, *HM* harmonic mean

Among the genes within 1 Mb of these selected markers, ontology terms were found to be significantly overrepresented in the gene lists based on DAVID in all comparisons (see Additional file 6: Table S3). When comparing between the two breeds, terms associated with inflammation (‘systemic lupus erythematosus’ and ‘inflammatory mediator regulation of TRP cells’) and histones (‘nucleosome’, ‘nucleosome core’, ‘histone-fold’, ‘histone core’ and ‘histone’) were detected. All other comparisons of groups had significant terms associated with various inflammatory and immune responses except for W3 against the other WB within-breed genetic groups, which had polar, acidic and basic residues as significant terms.

### Runs of homozygosity

**Fig. 7:**
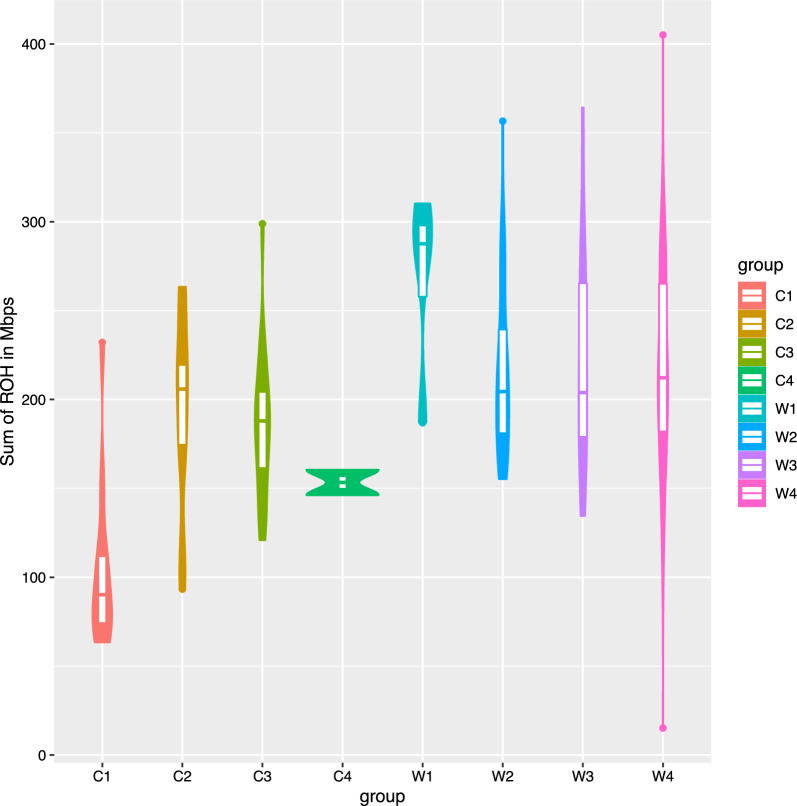
Violin plot illustrating sum length of runs of homozygosity (ROH) in the within-breed genetic groups of Connemara ponies (C1 to C4) and Warmblood horses (W1 to W4)

The W4 genetic group contained the individuals with both the largest and smallest sum of ROH lengths (total additive length of all calculated ROH), while the W1 genetic group (associated with Holsteiners) had the largest median sum of ROH lengths and the C1 genetic group (associated with non-registered CP) had the smallest (Fig. 7).

**Fig. 8:**
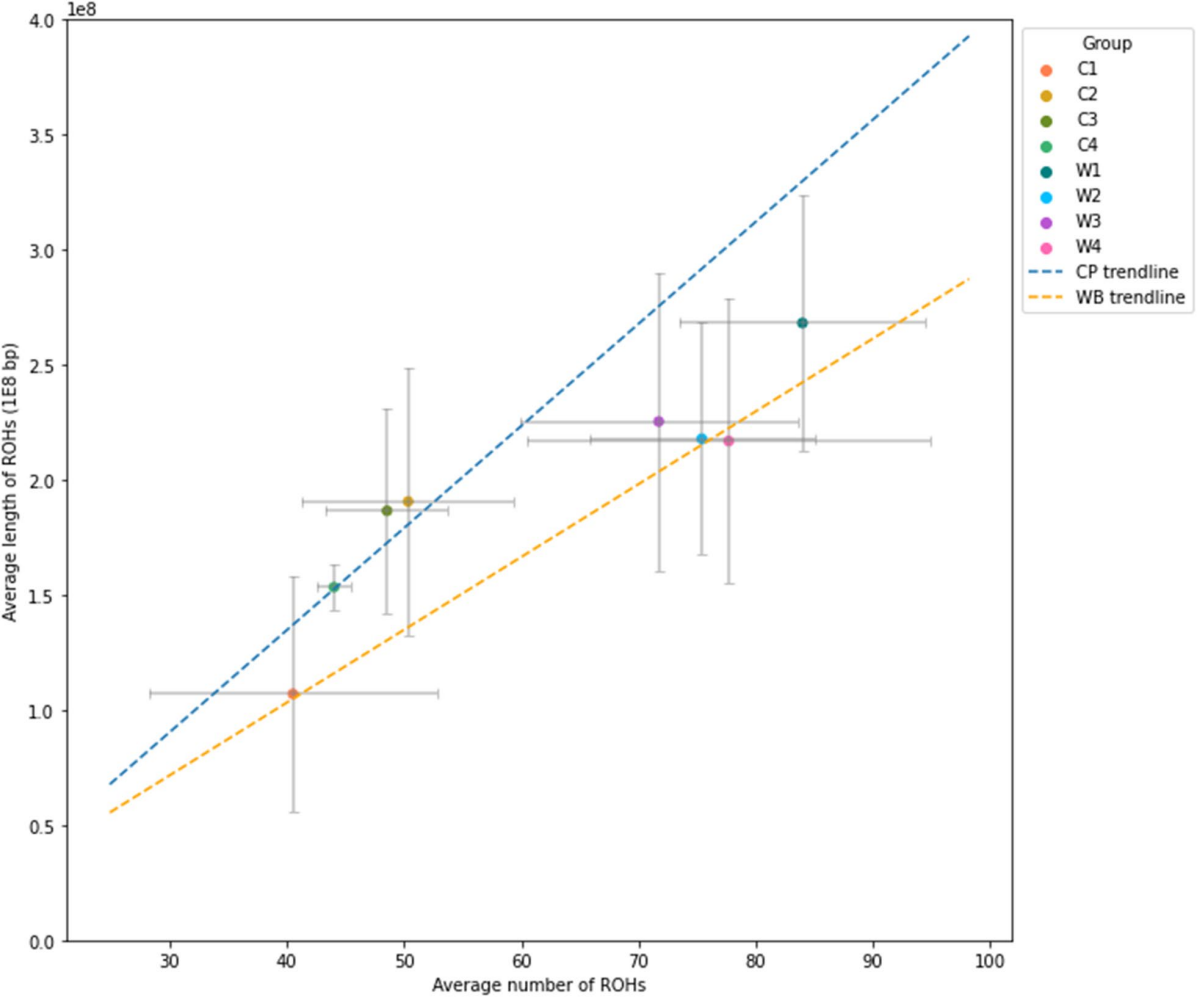
Mean length of runs of homozygosity (ROH) in CP and WB genetic groups compared with the mean number of ROH. Error bars represent standard deviation per group, and trendlines were calculated using ordinary least squares regression of all individuals from each breed. *CP* Connemara pony, *WB* Warmblood horse

CP genetic groups showed a smaller mean length of ROH and fewer ROH on average than the WB genetic groups (Fig. 8). This was a distinct breed difference, with the C1 genetic group tending to have the smallest sum of ROH lengths and smallest number of ROH amongst the CP genetic groups. Notably, the C2 and C3 genetic groups had an average length of ROH that was similar to that of the W2, W3 and W4 groups, although they had fewer ROH, and the slope of the regression line in CP was larger than in WB.

Overlapping ROH within breed, genetic group and origin group were identified (Additional file 7: Table S4). Genes within these ROH regions were analysed using DAVID, but no significantly overrepresented ontology terms were identified at the breed level (either unique to a given breed or shared by both). However, significant ontology terms were identified for some origin groups and within-breed genetic groups (see Additional file 8: Table S5). The C2 genetic group was predominantly associated with ontology terms for cell adhesion molecules, which were also identified in the genetic group analyses, while W1 was associated with ion channels and ion transport, and W2 with flavin adenine dinucleotide proteins that are involved in various redox reactions including the citric acid cycle. UK CP were associated with ontology terms for nitrogen metabolism, while European WB were associated with intermediate filaments, and keratin filaments.

### Genomic inbreeding

**Fig. 9:**
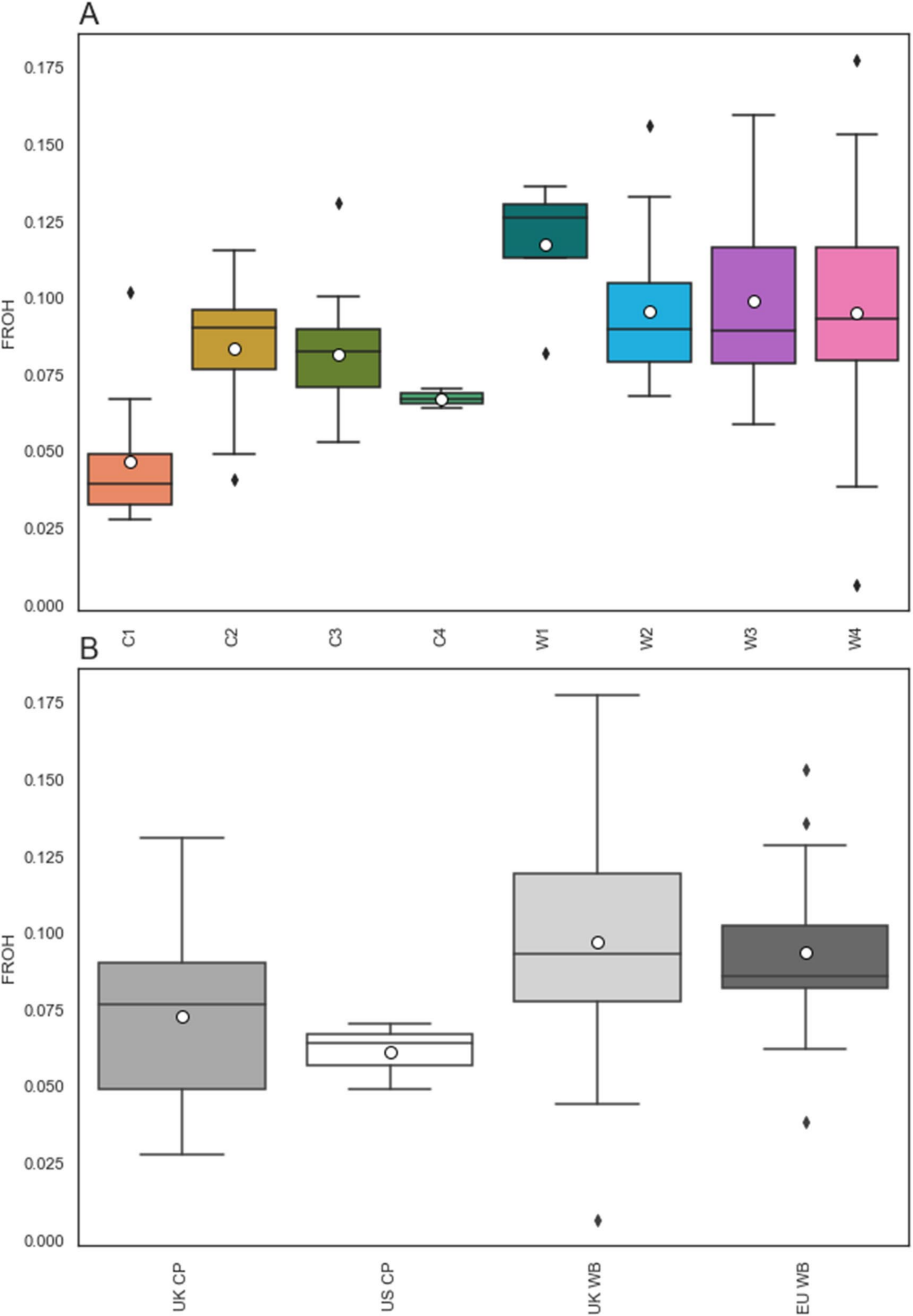
Boxplot of genomic inbreeding in CP and WB represented by F_ROH._ Boxplot of genomic inbreeding in CP and WB represented by F_ROH_, differentiated by: **A** within-breed genetic group; and **B** origin group. Mean is represented by white circles, median by a black line, and the box represents the second and third quartiles. Outliers (indicated by grey diamonds) are greater than 1.5 times the interquartile range from quartile 1 and quartile 3. *CP* Connemara pony, *WB* Warmblood horse

On average, F_ROH_ tended to be slightly lower in CP origin groups than in WB origin groups, with a mean of 0.073 and 0.061 in UK and US CP, respectively, compared to 0.097 and 0.094 in UK and EU WB (Fig. 9). When compared with one-way ANOVA, origin group had no significant impact on F_ROH_ within breed.

F_ROH_ was also examined across within-breed genetic groups. C1 had the lowest mean F_ROH_ (0.047) and W1 the highest (0.118), with a wider range of mean F_ROH_ values observed among WB within-breed genetic groups than among CP within-breed genetic groups (0.095–0.118 compared with 0.047–0.084). F_ROH_ differed significantly between genetic groups in CP (one-way ANOVA, p = 0.002) but not in WB (p = 0.40). Significant differences were identified between genetic groups C1 and C2 as well as between C1 and C3, using post hoc Tukey's testing.

When F_ROH_ was broken down to the per chromosome level, distinct distribution patterns began to emerge (See Additional file 9: Fig. S3). The highest mean F_ROH_ was observed for ECA25 in C1 and C2, but not in C3 (for which it was highest for ECA24) and C4 (highest for ECA8, 18 and 24). C4 had no ROH at all on ECA21, 22 and 27. W2 and W4 had a very even distribution of inbreeding along all the chromosomes, while the highest F_ROH_ observed for ECA12 in W3 and for ECA14 and 30 in W1, with no ROH on ECA29.

**Fig. 10:**
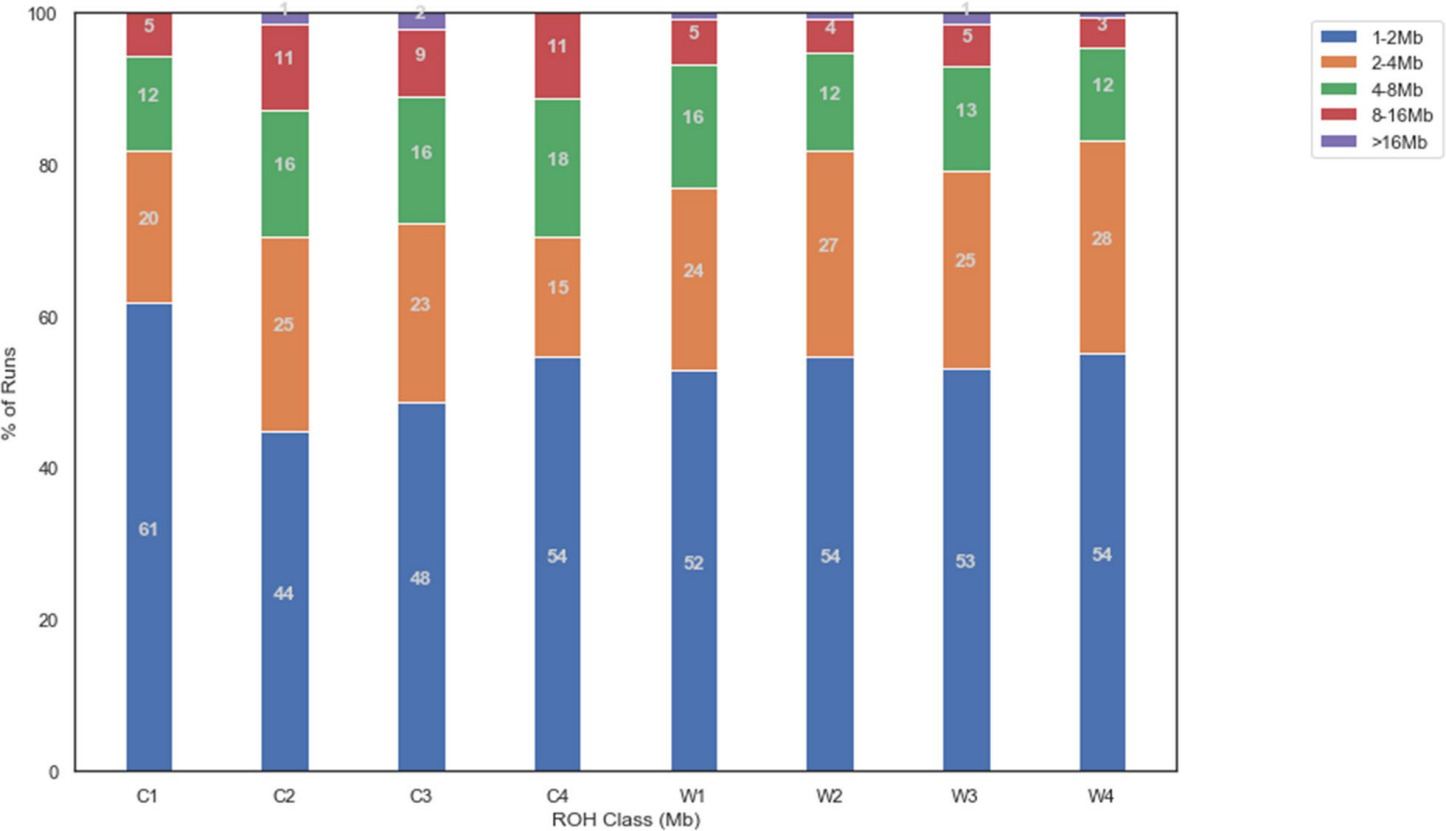
Percentage of runs of homozygosity (ROH) in within-breed genetic groups by ROH size class

When ROH were split by size class, it was noted that C2, C3 and C4 genetic groups had a greater proportion of runs longer than 4 Mb than the other genetic groups (Fig. 10). C2, C3 and W3 had the largest proportions of ROH longer than 16 Mb, while the genetic groups C1 and C4 had no ROH longer than 16 Mb. This implies that the genetic groups containing the registered CP (C2 to 4) have a greater degree of recent inbreeding than the within-breed WB and C1 genetic groups due to this greater proportion of large ROH.

## Discussion

The aim of this study was to characterise the genetic profiles of the little-studied Connemara pony breed and the well-documented Warmblood horse. These two breeds are very distinct with different origins, although both are now selected for performance in equestrian sport. Multiple genetic metrics were calculated and compared, including clustering analysis based on genotypic data; LD decay and LD block size distribution; N_e_ and N_eb_; ROH and F_ROH_. We found that the genetic substructure in the WB population was not associated with traditional subtypes (registered studbook), and that WB genetic groups tended to be, although not significantly, more inbred than registered CP genetic groups. We also identified a possible population structure in the CP population. While the number of US CP was too small to draw strong conclusions regarding geographical location, the basis of the separation of the remaining two UK-based, registered, non-admixed clusters could potentially be associated with various factors not analysed here including differences between breeding lines, breeder preferences, or diverging breeding goals. Both registered and unregistered CP genetic groups appeared to have a degree of popular sire choice comparable to that for WB based on the ratio of N_eb_ to N_e_, as well as indicators of a greater degree of recent inbreeding.

CP separated well from WB in the PCA, as might be expected for distinct breeds. Previous studies that compared WB and Scottish Highland ponies, which are among the closest related breeds to CP, also found that the breeds were very distinct [3], as well as studies that compared small numbers of WB and CP (n = 16 and n = 4, respectively) [85]. However, the current study identified few significant ontology terms between CP and WB in the analyses, mainly comprising a combination of histone-related terms and inflammatory terms. These findings contrast with previous comparisons of WB with non-sport breeds [85, 86], which identified terms associated with morphology and development. In addition, the terms identified between breeds were not any more related to performance than within-breed analyses, supporting the hypothesis that selection has gradually turned the CP into a sports breed.

For WB, in spite of the existence of many different WB subtypes associated with different studbooks, there was, in fact, little genomic differentiation between these subtypes, which implies that it is unlikely that the population sub-structure observed in the WB breed is due the historically location-based studbook of registration. Artificial insemination (AI) has been popular in the WB since the 1990’s, with varying levels of uptake in different countries depending on the managing studbook and availability of AI centres [87]. The German Equestrian Federation reported 30,491 coverings of WB in 2022, of which 29,174 were AI (27,140 fresh semen inseminations, 1047 frozen semen inseminations, and 987 embryo transfers) [31]. It is possible that, with modern breeding practices including the international travel of mares and shipping of semen [23, 88], location is less linked to specific WB lines than in the past, and therefore location does not accurately correlate with population structure. In contrast, non-registered CP associated with one genetic group (C1), and two of the three US CP with another (C4). Feely et al. [89] found a difference in relationship coefficients from pedigree data between Irish CP and six other worldwide regional populations, including from North America, which indicates some divergence and a source of genetic diversity in non-Irish populations. Although we only had three US CP in our study and therefore cannot draw strong conclusions on geographical effect, the separation that we observe could be explained by the findings of Feely et al. [89].

The lack of correlation between genetic group and breed subtype found in WB raises the question about whether genetic studies in WB should move away from the traditional use of breed subtype and registered studbook to describe population structure. Usage of genetic clustering as an alternative could reflect more closely the practice of cross-subtype use of particular sires. The presence of these genetic groups is evident in the results of previous phylogenetic, neighbour joining-tree and PCA studies of WB, where WB subtypes often appear in mixed clusters or clades, with some WB more closely related to Thoroughbreds or Standardbreds and others to Arabians or draught breeds [3, 85, 90–92].

A previous study of estimated breeding values for show jumping performance in Swedish Warmbloods demonstrated a clear genetic divergence between animals bred for show jumping versus dressage within subtype [22]. It is possible that other metrics, such as the specific discipline goal the horse is bred for, may prove more useful in subtyping WB horses than the registered studbook. Although we had access to data on the current discipline of approximately half of the animals in the study, due to the likely lack of direct correlation between current discipline and breeding goals we chose not to include this in our analysis. Traditionally, the UK has focused more on eventing than other European countries, which requires more stamina than show jumping and dressage and benefits from a lighter build [93]. Consequently, the Thoroughbred has been highly influential in British sport horse breeding. Thus, discipline could be one area in which the breeding goals of the Anglo European, British Warmblood and Holsteiner studbooks vary. However, the explicit grading requirements of the Anglo European Studbook [94], the British Warmblood Society [95, 96], and the Holsteiner Verband [97, 98] are reasonably similar, with both morphological and movement traits assessed in-hand, and a performance requirement – the former can be either a ridden jumping test or a dressage test in stallions [94], while the latter two require loose jumping in both stallions and mares [95–98]. Thus, selection preferences regarding discipline could be more culturally implicit than explicit within the breeding goals of these studbooks.

It is unclear why the Holsteiners would separate more than the Trakehners from other WB subtypes. Trakehners have a defined, closed studbook and are therefore expected to be the only genetically distinct WB subtype. Previous studies have revealed less overlap between Holsteiners and other German WB subtypes [29] than the Trakehners. Holsteiners have been described as having a “small nucleus of broodmares” compared with other German WB studbooks [21], anecdotally resulting in what the industry colloquially refers to as a particular ‘stamp’ or ‘type’. This refers to a physically recognisable appearance specific to the Holsteiner. This effect could be what we captured in the genetic analyses, however, morphologically, the Trakehner is also often described as resembling more closely the Thoroughbred than other WB subtypes, and no similar effect was seen with that subtype. However, ion channels and ion transport were identified as significantly associated with common ROH in genetic group W1 (pertaining to Holsteiners). Furthermore, intermediate filaments, which are important cytoskeletal components of myofibrils and connective tissues, were associated with EU WB (also pertaining to Holsteiners). This indicates that there is still genetic evidence of selection in different WB genetic groups, both historically for cavalry use and more recently for athletic performance [23].

Separation of Anglo European WB and British WB from the continental European studbooks in the PCA could be due to the common UK practice of breeding Irish Draught horses with Thoroughbreds to produce WB-like Irish Sports Horses predominantly for eventing. This could have affected the UK-based WB stock. The WB in the PCA that were located closest to CP did in fact have some Irish Sports Horses in their pedigrees. This reflects the historical influence of Irish Draughts on the CP, although such pedigree information was not available for all Anglo European and British WB to confirm this hypothesis. Furthermore, the historical reluctance of UK breeders to engage with the grading and registration procedures that are a core tenet of WB breeding and studbook registration in continental Europe [21] would likely place different selection pressures on UK horses than those from continental European studbooks. This may also contribute to genetic divergence in UK-based studbooks.

The results of the LD patterns across all origin groups and within-breed genetic groups showed lower values than previously reported in Thoroughbreds [99], but similar to previous across-breed values [100] and to reports within a range of different horse breeds [3] as well as with LD calculations in WB specifically [85]. While LD presented a slower decay in CP than in WB within the first four Mb, the peak of the LD block size distributions was the same in both breeds, indicating that LD blocks of up to 1 Mb are quite common in both breeds. Variation in mean LD was also large between within-breed genetic groups. In comparison, the origin groups (which encompassed multiple genetic groups) showed deflated means and maximal LD. This supports the conclusions that these within-breed genetic groups are likely genetically distinct subpopulations. North American CP were distinct from Irish and UK CP in a previous pedigree-based study [89]. This could explain the differences observed for some of the results in the C4 group. However, the small numbers of US CP and the small size of C4 did limit the inclusion of this genetic group in some analyses.

Estimates of effective population size were carried out within specific genetic groups, and specifically those of similar sample size. A similar N_eb_/N_e_ ratio was found between the W3 genetic group and the median of the CP genetic groups with similar sample size (C1, C2, and C3). A lower ratio can be indicative of a skewed ratio of breeding stallions to mares, indicating that popular sires are contributing to the gene pool to a greater degree. While some studies indicate that WB are affected by the choice of popular sires [21, 101], the W3 group was mainly associated with British and Anglo European WB, and it is possible that this group does not accurately represent the degree of popular sire choice in continental European subtypes. For CP, two of the three within-breed genetic groups (C1 and C3) had an equal or lower ratio to W3, indicating an equal or greater degree of popular sire choice in CP. This finding is supported by pedigree studies on CP where selection of popular sires was important [89, 102].

In spite of the similar or greater degree of popular sire choice, registered CP tended to be, although not significantly, less inbred than WB, with the non-registered CP significantly less inbred than the CP of all groups but C4—most likely due to admixture. To our knowledge, our study is the first to estimate genomic inbreeding using the F_ROH_ method in CP, so comparisons with previous studies based on pedigree estimates are difficult [103], e.g.. studies in Italian Heavy Draught horses [104], Norwegian-Swedish Coldblooded Trotters [105], and Sztumski and Sokólski horses [106] found F_ROH_ to be much higher than pedigree estimates. Specifically, in the study of Feely et al. [89], estimates of the mean inbreeding coefficient from pedigree data are equal to 0.047, 0.044 and 0.040 in Irish, UK and North American CP, respectively, which are lower than those based on genomic data in UK and US CP in the present study. This could simply indicate that F_ROH_ does tend to be higher than pedigree estimates, or also a recent increase in inbreeding.

The inbreeding values that we found for CP are not particularly high compared to those for rare North European breeds [107], but a recent increase in inbreeding could still be a cause for concern. While yearly average pedigree inbreeding values as high as 0.11 have been reported in Welsh ponies, no notable increase in inbreeding was observed between 1970 and 2014 [108], indicating that inbreeding in that breed is well managed. On the contrary, a steady increase in pedigree inbreeding values was reported in CP between 1980 to 2000 [102] at a rate similar to that expected under random mating and without selecting for non-related animals. There are also previous findings showing that genetic diversity in CP is decreasing over time [89], which is consistent with evidence from our study, although one must keep in mind that genetic and pedigree-based inbreeding are not necessarily comparable. CP inbreeding calculated from expected heterozygosity has also been directly compared to the four Welsh Studbook Sections (A: Welsh Mountain Pony; B: Welsh Pony of Riding Type; C: Welsh Pony of Cob Type; D: Welsh Cob), and was found to be within a similar range (CP: 0.033; A: 0.033; B: 0.020; C: 0.049; D: 0.017) [18]. Furthermore, a larger proportion of longer ROH in registered CP-associated genetic groups than in WB genetic groups was identified, which indicates more recent inbreeding.

Furthermore, differences in signatures of selection were identified between breeds as well as between within-breed genetic groups. The immune and inflammatory ontology terms identified in all within-breed genetic groups in the F_ST_ analysis were reported in a previous study on exercising horses [109] that also detected apoptotic [110, 111] and inflammatory pathways [112–115] related to exercise-induced oxidative stress response [116]. In a study on signatures of selection between ‘primitive’ and ‘light’ horse breeds, immune system functions were the most enriched [117]. Immune terms are also associated with exercise in the horse [115], with immune and inflammatory genes typically upregulated, likely due to exercise-induced muscle damage [118, 119]. These significant immune terms were present in every within-breed comparison, with immunoglobulin or antibody terms appearing in every within-breed comparison except W3, which could indicate that differentiation between W3 and the other WB genetic groups is too broad to be associated to one particular pathway. As the genetic group clustering was carried out using principal components from PCA, it is possible that the highly polymorphic nature of the immune system genes [120] may have contributed to genetic group allocation. However, there were fewer immune related terms in the between-breed analysis, with more ontology terms associated with histones.

## Conclusions

In conclusion, the genetic characterisation of the CP and WB has identified several key findings. The genetic variation and population substructure in the WB is not well captured by subtype based on the registered studbook and it is likely that a similar genetic effect of popular sire choice is present in the CP as in the WB, which is thought to be considerable. We report the first estimates of inbreeding from ROH in CP, and found that CP have a similar or slightly lower average level of inbreeding than WB but with a greater degree of recent inbreeding. Hopefully, these findings will prompt further studies to better understand the population substructure in WB horses, and act as an early warning to breeders of CP that proactive changes in breed management are required to sustain genetic variation and overall breed health in this highly popular breed.

### Supplementary Information

**Additional file 1: Methods S1.** Additional methods: DNA extraction protocols. Protocols used for DNA extraction from equine muscle, blood and hair root samples

**Additional file 2:Table S1.** Horse breed and sample type**.** Table indicating the breed, breed subtype, sample origin, sample type and genotyping platform of each sample. CP: Connemara pony; WB: Warmblood horse; KWPN: Koninklijk Warmbloed Paardenstamboek Nederland; M: male; F: female; WGS: whole genome sequencing; SNP: single nucleotide polymorphism array genotyping panel.

**Additional file 3: Figure S1.** Elbow plot using within-sum of squares and silhouette plot for selection of appropriate number of clusters**.** Elbow plots (top) and silhouette plots (bottom) for selection of appropriate number of clusters (k) for k-means clustering analysis in WB (left) and CP (right). CP: Connemara pony; WB: Warmblood horse; WSS: within sum of squares; k: number of clusters.

**Additional file 4: Figure S2.** Principal components (PCs) of the genetic relationship matrices for 116 WB (lower diagonal) and 36 CP (upper diagonal). Principal components of the genetic relationship matrices for 116 WB (lower diagonal) and 36 CP (upper diagonal). Upper and lower diagonal plots for CP and WB respectively, with colour designating the k-means assigned cluster and marker designating breed subtype: in CP B) principal component (PC) 1 by PC 2; C) PC 1 by PC 3; and F) PC 2 by PC 3; in WB D) PC 1 by PC 2; G) PC 1 by PC 3; and H) PC 2 by PC 3. Diagonal plots are kernel density estimator plots illustrating the distribution of the principal components, with distribution curves for each cluster: A) PC 1; E) PC 2; and I) PC 3. The first three PC in CP explained 4.7%, 4.2% and 3.7% of variance respectively, and in WB explained 2.5%, 2.2% and 1.7% of variance respectively. CP: Connemara pony; WB: Warmblood horse; UK: United Kingdom; EU: rest of Europe; US: United States; X: unregistered.

**Additional file 5: Table S2.** Over-representation of breed subtypes and sample origin in CP and WB within-breed genetic groups**.** Breed subtypes and sample origins significantly associated with different genetic groups using Chi-square testing. CP: Connemara pony; WB: Warmblood horse; UK: United Kingdom; EU: rest of Europe; US: United States; X: unregistered.

**Additional file 6: Table S3.** Significant terms associated with genes within 1 Mb of top 0.5% markers. Ontology terms identified as significantly over-represented in genes within 1 Mb of the top 0.5% SNPs between different groups using DAVID. CP: Connemara pony; WB: Warmblood horse; F_ST_: Wright’s fixation index.

**Additional file 7: Table S4.** Largest number of individuals and % of the group sharing a run of homozygosity (ROH) in CP and WB, and in within-breed genetic groups. CP: Connemara pony; WB: Warmblood horse; ROH: runs of homozygosity.

**Additional file 8: Table S5**. Significant ontology terms associated with genes within ROH regions present in > 10% of horses belonging to a given genetic group, origin group or breed. Ontology terms identified as significantly over-represented in genes within 1 Mb of the ROH regions present in > 10% of horses belonging to a given genetic group, origin group or breed using DAVID. CP: Connemara pony; WB: Warmblood horse; ROH: runs of homozygosity.

**Additional file 9: Figure S3.** Mean genomic inbreeding (as F_ROH_) by chromosome, illustrated in within-breed genetic group**.** Mean genomic inbreeding (as F_ROH_) by chromosome, illustrated in within-breed genetic group. CP: Connemara pony; WB: Warmblood horse; ROH: runs of homozygosity.

## Data Availability

The datasets used and/or analysed during the current study are available from the corresponding author on reasonable request.

## References

[1] Charlesworth D, Willis JH (2009). The genetics of inbreeding depression. Nat Rev Genet.

[2] Khadka R. Global horse population with respect to breeds and risk status. Master thesis, Swedish University of Agricultural Sciences; 2010.

[3] Petersen JL, Mickelson JR, Cothran EG, Andersson LS, Axelsson J, Bailey E (2013). Genetic diversity in the modern horse illustrated from genome-wide SNP data. PLoS One..

[4] Westemeier RL, Brawn JD, Simpson SA, Esker TL, Jansen RW, Walk JW (1998). Tracking the long-term decline and recovery of an isolated population. Science.

[5] Mac Lochlainn T (2021). The Connemara pony: a history.

[6] Lyne P (1984). Shrouded in mist: the Connemara pony.

[7] Petch E (1998). Connemara pony breeders' society, 1923–1998.

[8] Brown CJ, Davis DL, Maurstad A (2016). From working to winning: the shifting symbolic value of Connemara ponies in the West of Ireland. The meaning of horses Biosocial Encounters.

[9] O'Hare N. Great Connemara Stalions. Harkaway, Co. Meath Ireland; 2008.

[10] British Connemara Pony Society. British Connemara Pony Society Stud Book. 2019. https://www.britishconnemaras.co.uk/. Accessed 08 Mar 2023.

[11] Rare Breeds Survival Trust. Watchlist 2021–22: Rare Breeds Survival Trust. 2021 https://www.rbst.org.uk/rbst-watchlist/. Accessed 27 Sep 2022.

[12] Finno CJ, Stevens C, Young A, Affolter V, Joshi NA, Ramsay S (2015). SERPINB11 frameshift variant associated with novel hoof specific phenotype in Connemara ponies. PLoS Genet.

[13] Connemara Pony Breeders' Society. The Connemara Pony Breeders’ Society Breeding Programme. 2020. https://cpbs.ie/wp-content/uploads/2022/01/Connemara-Pony-Breeders-Society-Breeding-Programme-.pdf/. Accessed 08 Mar 2023.

[14] British Connemara Pony Society. Hoof wall separation disease. 2023. https://www.britishconnemaras.co.uk/breeding-owning/hwsd/. Accessed 08 Mar 2023.

[15] McGahern A, Edwards CJ, Bower M, Heffernan A, Park S, Brophy P (2006). Mitochondrial DNA sequence diversity in extant Irish horse populations and in ancient horses. Anim Genet.

[16] Winton CL, Hegarty MJ, McMahon R, Slavov GT, McEwan NR, Davies-Morel MC (2013). Genetic diversity and phylogenetic analysis of native mountain ponies of Britain and Ireland reveals a novel rare population. Ecol Evol.

[17] Khanshour AM, Hempsey EK, Juras R, Cothran E (2019). Genetic characterization of Cleveland bay horse breed. Diversity.

[18] Winton CL, McMahon R, Hegarty MJ, McEwan NR, Davies-Morel MC, Morgan C (2020). Genetic diversity within and between British and Irish breeds: the maternal and paternal history of native ponies. Ecol Evol.

[19] Pritchard JK, Stephens M, Donnelly P (2000). Inference of population structure using multilocus genotype data. Genetics.

[20] Bower MA, Campana MG, Whitten M, Edwards CJ, Jones H, Barrett E (2011). The cosmopolitan maternal heritage of the Thoroughbred racehorse breed shows a significant contribution from British and Irish native mares. Biol Lett.

[21] Wallin D, Kidd J, Clarke C (1995). The International Warmblood horse: a worldwide guide to breeding and bloodlines.

[22] Ablondi M, Eriksson S, Tetu S, Sabbioni A, Viklund Å, Mikko S (2019). Genomic divergence in Swedish Warmblood horses selected for equestrian disciplines. Genes (Basel).

[23] Koenen EPC, Aldridge LI, Philipsson J (2004). An overview of breeding objectives for warmblood sport horses. Livest Prod Sci.

[24] Stock K, Distl O (2007). Genetic correlations between performance traits and radiographic findings in the limbs of German Warmblood riding horses. J Anim Sci.

[25] Viklund Å, Braam Å, Näsholm A, Strandberg E, Philipsson J (2010). Genetic variation in competition traits at different ages and time periods and correlations with traits at field tests of 4-year-old Swedish Warmblood horses. Animal.

[26] Borowska A, Wolc A, Szwaczkowski T (2011). Genetic variability of traits recorded during 100-day stationary performance test and inbreeding level in Polish warmblood stallions. Arch Anim Breed.

[27] Schröder W, Klostermann A, Stock KF, Distl O (2012). A genome-wide association study for quantitative trait loci of show-jumping in Hanoverian warmblood horses. Anim Genet.

[28] Stewart ID, White IMS, Gilmour AR, Thompson R, Woolliams JA, Brotherstone S (2012). Estimating variance components and predicting breeding values for eventing disciplines and grades in sport horses. Animal.

[29] Nolte W, Thaller G, Kuehn C (2019). Selection signatures in four German warmblood horse breeds: Tracing breeding history in the modern sport horse. PLoS One..

[30] Eurodressage. German Equestrian Federation Discloses Breeding Statistics for 2018. 2018. https://www.eurodressage.com/2019/04/02/german-equestrian-federation-discloses-breeding-statistics-2018/. Accessed 27 Sep 2022.

[31] Deutsche Reiterliche Vereinigung (FN). Jahresbericht 2022 Bereich Zucht. 2022. https://www.pferd-aktuell.de/shop/broschuren-formulare-vertrage-unterrichtsmaterial/jahresberichte-fn-dokr.html/. Accessed 26 Apr 2023.

[32] Deutsche Reiterliche Vereinigung (FN), Deutches Olympiade-Komitee für Reiterei. Jahresbericht 2021. 2021. https://www.pferd-aktuell.de/deutsche-reiterliche-vereinigung/verbandsstruktur-der-fn/dokr-und-bundesstuetzpunkt/. Accessed 08 Mar 2023.

[33] Slater J. National Equine Health Survey; Blue Cross. 2016. https://www.bluecross.org.uk/national-equine-health-survey/. Accessed 27 Sep 2022.

[34] Slater J. National Equine Health Survey; Blue Cross. 2017. https://www.bluecross.org.uk/national-equine-health-survey//. Accessed 27 Sep 2022.

[35] Taylor G, Slater J. National Equine Health Survey; Blue Cross. 2018. https://www.bluecross.org.uk/national-equine-health-survey/. Accessed 27 Sep 2022.

[36] Heuer C, Scheel C, Tetens J, Kühn C, Thaller G (2016). Genomic prediction of unordered categorical traits: an application to subpopulation assignment in German Warmblood horses. Genet Sel Evol.

[37] Wright S (1931). Evolution in Mendelian populations. Genetics.

[38] Barbato M, Orozco-terWengel P, Tapio M, Bruford MW (2015). SNeP: a tool to estimate trends in recent effective population size trajectories using genome-wide SNP data. Front Genet.

[39] Jorde PE, Ryman N (1995). Temporal allele frequency change and estimation of effective size in populations with overlapping generations. Genetics.

[40] Nomura T (2008). Estimation of effective number of breeders from molecular coancestry of single cohort sample. Evol Appl.

[41] Alemu SW, Kadri NK, Harland C, Faux P, Charlier C, Caballero A (2021). An evaluation of inbreeding measures using a whole-genome sequenced cattle pedigree. Heredity (Edinb).

[42] Howrigan DP, Simonson MA, Keller MC (2011). Detecting autozygosity through runs of homozygosity: a comparison of three autozygosity detection algorithms. BMC Genomics.

[43] Keller MC, Visscher PM, Goddard ME (2011). Quantification of inbreeding due to distant ancestors and its detection using dense single nucleotide polymorphism data. Genetics.

[44] McQuillan R, Leutenegger A-L, Abdel-Rahman R, Franklin CS, Pericic M, Barac-Lauc L (2008). Runs of homozygosity in European populations. Am J Hum Genet.

[45] Schaefer RJ, Schubert M, Bailey E, Bannasch DL, Barrey E, Bar-Gal GK (2017). Developing a 670k genotyping array to tag~ 2M SNPs across 24 horse breeds. BMC Genomics.

[46] Van der Auwera GA, O'Connor BD. Genomics in the Cloud: Using Docker, GATK, and WDL in Terra. Sebastopol: O'Reilly Media; 2020.

[47] DePristo MA, Banks E, Poplin R, Garimella KV, Maguire JR, Hartl C (2011). A framework for variation discovery and genotyping using next-generation DNA sequencing data. Nat Genet.

[48] Purcell S, Neale B, Todd-Brown K, Thomas L, Ferreira MA, Bender D (2007). PLINK: a tool set for whole-genome association and population-based linkage analyses. Am J Hum Genet.

[49] Zhou X, Stephens M (2012). Genome-wide efficient mixed-model analysis for association studies. Nat Genet.

[50] Waskom ML (2021). seaborn: statistical data visualization. J Open Source Softw.

[51] Hunter JD (2007). Matplotlib: A 2D graphics environment. Comput Sci Eng.

[52] Kassambra A, Mundt F. factoextra: Extract and visualize the results of multivariate data analyses. R package version 1.0.7. 2020. https://CRAN.R-project.org/package=factoextra/. Accessed 27 Sep 2022.

[53] Seabold S, Perktold J. Statsmodels: Econometric and statistical modeling with python. In: Proceedings of the 9th Python in Science Conference: 28 June-3 July 2010; Austin; 2010.

[54] Howey R, Cordell HJ. Mapthin. 2011. http://www.staff.ncl.ac.uk/richard.howey/mapthin/. Accessed 27 Sep 2022.

[55] Wickham H, François R, Henry L, Müller K. dplyr: A Grammar of Data Manipulation. R package version 1.0.7 ed. 2021. https://CRAN.R-project.org/package=dplyr/. Accessed 27 Sep 2022.

[56] Wickham H. stringr: Simple, consistent wrappers for common string operations. R package version 1.4.0. 2019. https://CRAN.R-project.org/package=stringr/. Accessed 27 Sep 2022.

[57] Wickham H (2016). ggplot2: Elegant graphics for data analysis.

[58] Sved JA, Feldman MW (1973). Correlation and probability methods for one and two loci. Theor Pop Biol.

[59] Corbin LJ, Liu A, Bishop SC, Woolliams JA (2012). Estimation of historical effective population size using linkage disequilibria with marker data. J Anim Breed Genet.

[60] Pedregosa F, Varoquaux G, Gramfort A, Michel V, Thirion B, Grisel O (2011). Scikit-learn: Machine Learning in Python. J Mach Learn Res.

[61] Lischer HE, Excoffier L (2012). PGDSpider: an automated data conversion tool for connecting population genetics and genomics programs. Bioinformatics.

[62] Do C, Waples RS, Peel D, Macbeth G, Tillett BJ, Ovenden JR (2014). NeEstimator v2: re-implementation of software for the estimation of contemporary effective population size (Ne) from genetic data. Mol Ecol Resour.

[63] Goudet J (2005). Hierfstat, a package for R to compute and test hierarchical F-statistics. Mol Ecol Notes.

[64] Chang CC, Chow CC, Tellier LC, Vattikuti S, Purcell SM, Lee JJ (2015). Second-generation PLINK: rising to the challenge of larger and richer datasets. Gigascience.

[65] Virtanen P, Gommers R, Oliphant TE, Haberland M, Reddy T, Cournapeau D (2020). SciPy 10: Fundamental algorithms for scientific computing in Python. Nat Methods.

[66] Turner SD (2018). qqman: an R package for visualizing GWAS results using Q-Q and manhattan plots. J Open Source Softw.

[67] Durinck S, Moreau Y, Kasprzyk A, Davis S, De Moor B, Brazma A (2005). BioMart and Bioconductor: a powerful link between biological databases and microarray data analysis. Bioinformatics.

[68] Durinck S, Spellman PT, Birney E, Huber W (2009). Mapping identifiers for the integration of genomic datasets with the R/Bioconductor package biomaRt. Nat Protoc.

[69] Dennis G, Sherman BT, Hosack DA, Yang J, Gao W, Lane HC (2003). DAVID: database for annotation, visualization, and integrated discovery. Genome Biol.

[70] Ashburner M, Ball CA, Blake JA, Botstein D, Butler H, Cherry JM (2000). Gene ontology: tool for the unification of biology. Nat Genet.

[71] Kanehisa M, Goto S (2000). KEGG: kyoto encyclopedia of genes and genomes. Nucleic Acids Res.

[72] Kanehisa M (2019). Toward understanding the origin and evolution of cellular organisms. Protein Sci.

[73] Kanehisa M, Furumichi M, Sato Y, Ishiguro-Watanabe M, Tanabe M (2021). KEGG: integrating viruses and cellular organisms. Nucleic Acids Res.

[74] Gene Ontology Consortium (2021). The Gene Ontology resource: enriching a GOld mine. Nucleic Acids Res.

[75] Biscarini F, Cozzi P, Gaspa G, Marras G. detectRUNS: Detect runs of homozygosity and runs of heterozygosity in diploid genomes. R package version 0.9.6. 2019. https://CRAN.R-project.org/package=detectRUNS/. Accessed 27 Sep 2022.

[76] Meyermans R, Gorssen W, Buys N, Janssens S (2020). How to study runs of homozygosity using PLINK? A guide for analyzing medium density SNP data in livestock and pet species. BMC Genomics.

[77] Nothnagel M, Lu TT, Kayser M, Krawczak M (2010). Genomic and geographic distribution of SNP-defined runs of homozygosity in Europeans. Hum Mol Genet.

[78] Quinlan AR, Hall IM (2010). BEDTools: a flexible suite of utilities for comparing genomic features. Bioinformatics.

[79] Quinlan AR (2014). BEDTools: the Swiss-army tool for genome feature analysis. Curr Protoc Bioinformatics..

[80] Smedley D, Haider S, Ballester B, Holland R, London D, Thorisson G (2009). BioMart—biological queries made easy. BMC Genomics.

[81] Schiavo G, Bovo S, Bertolini F, Tinarelli S, DallOlio S, Costa LN (2020). Comparative evaluation of genomic inbreeding parameters in seven commercial and autochthonous pig breeds. Animal.

[82] Jones AT, Ovenden JR, Wang YG (2016). Improved confidence intervals for the linkage disequilibrium method for estimating effective population size. Heredity (Edinb).

[83] Waples RS (2015). Testing for Hardy-Weinberg proportions: Have we lost the plot?. J Hered.

[84] Nei M (1973). Analysis of gene diversity in subdivided populations. Proc Natl Acad Sci USA.

[85] SalekArdestani S, Aminafshar M, ZandiBaghcheMaryam MB, Banabazi MH, Sargolzaei M, Miar Y (2020). Whole-genome signatures of selection in sport horses revealed selection footprints related to musculoskeletal system development processes. Animals (Basel)..

[86] Metzger J, Karwath M, Tonda R, Beltran S, Águeda L, Gut M (2015). Runs of homozygosity reveal signatures of positive selection for reproduction traits in breed and non-breed horses. BMC Genomics.

[87] Aurich JE (2012). Artificial insemination in horses—more than a century of practice and research. J Eq Vet Sci.

[88] Langlois B, Blouin C (2004). Statistical analysis of some factors affecting the number of horse births in France. Reprod Nutr Dev.

[89] Feely D, Brophy P, Quinn K, Bodó I, Alderson L, Langlois B (2005). Characterisation of several Connemara Pony populations. Conservation genetics of endangered horse breeds The European Association for Animal Production Scientific Series.

[90] Glowatzki-Mullis M, Muntwyler J, Pfister W, Marti E, Rieder S, Poncet P (2006). Genetic diversity among horse populations with a special focus on the Franches-Montagnes breed. Anim Genet.

[91] Grilz-Seger G, Neuditschko M, Ricard A, Velie B, Lindgren G, Mesarič M (2019). Genome-wide homozygosity patterns and evidence for selection in a set of European and near eastern horse breeds. Genes (Basel).

[92] Schurink A, Shrestha M, Eriksson S, Bosse M, Bovenhuis H, Back W (2019). The gGenomic makeup of nine horse populations smpled in the Netherlands. Genes (Basel).

[93] Dyson S (2002). Lameness and poor performance in the sport horse: dressage, show jumping and horse trials. J Eq Vet Sci.

[94] Anglo European Studbook. Grading Procedures 2023. https://angloeuropeanstudbook.com/information/grading-procedures/. Accessed 22 Mar 2023.

[95] The Warmblood Breeders' Studbook UK. Stallion Grading 2023. https://bwbs.co.uk/info2.cfm?info_id=222213//. Accessed 22 Mar 2023.

[96] The Warmblood Breeders' Studbook UK. Mare Grading 2023. https://bwbs.co.uk/info2.cfm?info_id=222212/. Accessed 22 Mar 2023.

[97] Holsteiner Verband. Stallions 2023. https://www.holsteiner-verband.de/en/verband/Hengste/. Accessed 22 Mar 2023.

[98] Holsteiner Verband. Holsteiner Mares 2023. https://www.holsteiner-verband.de/en/verband/stuten/. Accessed 22 Mar 2023.

[99] Corbin LJ, Blott S, Swinburne J, Vaudin M, Bishop SC, Woolliams JA (2010). Linkage disequilibrium and historical effective population size in the Thoroughbred horse. Anim Genet.

[100] Wade C, Giulotto E, Sigurdsson S, Zoli M, Gnerre S, Imsland F (2009). Genome sequence, comparative analysis, and population genetics of the domestic horse. Science.

[101] Próchniak T, Kasperek K, Knaga S, Rozempolska-Rucińska I, Batkowska J, Drabik K (2021). Pedigree analysis of warmblood horses participating in competitions for young horses. Front Genet.

[102] Feely D, Brophy P, Quinn K (2003). Characterisation of the Connemara pony population in Ireland.

[103] VanRaden PM, Olson KM, Wiggans GR, Cole JB, Tooker ME (2011). Genomic inbreeding and relationships among Holsteins, Jerseys, and Brown Swiss. J Dairy Sci.

[104] Mancin E, Ablondi M, Mantovani R, Pigozzi G, Sabbioni A, Sartori C (2020). Genetic variability in the Italian heavy draught horse from pedigree data and genomic information. Animals (Basel).

[105] Velie BD, Solé M, Fegraeus KJ, Rosengren MK, Røed KH, Ihler C-F (2019). Genomic measures of inbreeding in the Norwegian-Swedish Coldblooded Trotter and their associations with known QTL for reproduction and health traits. Genet Sel Evol.

[106] Polak G, Gurgul A, Jasielczuk I, Szmatoła T, Krupiński J, Bugno-Poniewierska M (2021). Suitability of pedigree information and genomic methods for analyzing inbreeding of Polish cold-blooded horses covered by conservation programs. Genes (Basel).

[107] Saastamoinen M, Maenpaa M, Bodó I, Alderson L, Langlois B (2005). Rare horse breeds in Northern Europe. Conservation genetics of endangered horse breeds. The European Association for Animal Production Scientific Series.

[108] McMahon R, Debbonaire A, McEwan N, Nash D, Davies-Morel M, Winton C (2015). Report prepared for the WPCS-2015: a preliminary examination of the genetic variation within and between the improvement society herds of Welsh Mountain ponies.

[109] Park W, Kim J, Kim HJ, Choi J, Park J-W, Cho H-W (2014). Investigation of de novo unique differentially expressed genes related to evolution in exercise response during domestication in Thoroughbred race horses. PLoS One..

[110] Gourlay CW, Ayscough KR (2005). The actin cytoskeleton: a key regulator of apoptosis and ageing?. Nat Rev Mol Cell Biol.

[111] Saleem A, Adhihetty PJ, Hood DA (2009). Role of p53 in mitochondrial biogenesis and apoptosis in skeletal muscle. Physiol Genomics.

[112] Niess A, Dickhuth H, Northoff H, Fehrenbach E (1999). Free radicals and oxidative stress in exercise–immunological aspects. Exerc Immunol Rev.

[113] Dousset E, Avela J, Ishikawa M, Kallio J, Kuitunen S, Kyrolainen H (2007). Bimodal recovery pattern in human skeletal muscle induced by exhaustive stretch-shortening cycle exercise. Med Sci Sports Exerc.

[114] Andersson L (2012). How selective sweeps in domestic animals provide new insight into biological mechanisms. J Intern Med.

[115] Kim H, Lee T, Park W, Lee JW, Kim J, Lee B-Y (2013). Peeling back the evolutionary layers of molecular mechanisms responsive to exercise-stress in the skeletal muscle of the racing horse. DNA Res.

[116] Kingston SG, Hoffman-Goetz L (1996). Effect of environmental enrichment and housing density on immune system reactivity to acute exercise stress. Physiol Behav.

[117] Gurgul A, Jasielczuk I, Semik-Gurgul E, Pawlina-Tyszko K, Stefaniuk-Szmukier M, Szmatoła T (2019). A genome-wide scan for diversifying selection signatures in selected horse breeds. PLoS One..

[118] Cannon JG, St Pierre BA (1998). Cytokines in exertion-induced skeletal muscle injury. Mol Cell Biochem.

[119] Clarkson PM, Sayers SP (1999). Etiology of exercise-induced muscle damage. Can J Appl Physiol.

[120] Kwok AJ, Mentzer A, Knight JC (2021). Host genetics and infectious disease: new tools, insights and translational opportunities. Nat Rev Genet.

