# Supplementary material for: Genetic characterisation of the Connemara pony and the Warmblood horse using a within-breed clustering approach
Source: Genet Sel Evol. 2023 Aug 17;55:60. doi: 10.1186/s12711-023-00827-w (PMC10436415; doi:10.1186/s12711-023-00827-w)
Supplement: Supplementary file 1 — Additional file 1: Methods S1. Additional methods: DNA extraction protocols. Protocols used for DNA extraction from equine muscle, blood and hair root samples [file 12711_2023_827_MOESM1_ESM.docx]

**Additional file 1: Methods S1**

**DNA extraction protocol from horse muscle using the Qiagen DNEasy Blood & Tissue kit**

Before starting:

• All centrifugation steps are carried out at room temperature (15–25°C) in a

• micro centrifuge.

• Vortexing should be performed by pulse-vortexing for 5–10 s.

• Buffer AL may form a precipitate upon storage. If necessary, warm to 56°C

until the precipitate has fully dissolved.

• Buffer AW1 and Buffer AW2 are supplied as concentrates. Before using for

the first time, add the appropriate amount of ethanol (96–100%) as indicated

on the bottle to obtain a working solution.

• Preheat a thermomixer, shaking water bath, or rocking platform to 56°C for

use in step 2.

***Method***

1. Cut approximately 10-20 20 micron sections from an OCT-mounted muscle sample using a cryostat. Add sections to a dry ice-chilled 2 mL microcentrifuge tube containing a Tissuelyser bead. Clean the blade, roll bar and tweezers with ethanol between each sample. Each muscle sample should be sampled in duplicate.
2. Place sample tubes into a Qiagen Tissuelyser LT adapter. Add 200 mL of Qiagen Buffer ATL, close adapter lid, and sit for 2 minutes.
3. Run the Tissuelyser for 1 minute at 30 Hertz.
4. Add 20 μl of proteinase K and incubate at 56 °C for 1 hour.
5. Add 200 μl Buffer AL and a further 20 μl of proteinase K. Mix thoroughly by vortexing, and incubate at 56°C for 2 hours. Vortex samples every 15 mins.
6. Add 300 μl ethanol (96–100%) to the sample, and mix thoroughly by vortexing. It is important that the sample and the ethanol are mixed thoroughly to yield a homogeneous solution. Leave for 30 mins at room temperature.
7. Pipette half (370 μl) the solution from step 3 into the DNeasy Mini spin column placed in a 2-ml collection tube (provided). Centrifuge at 6000 x g (8000 rpm) for 2min. Discard flow-through and collection tube. Repeat step 7 with the other half of the solution.
8. Place the DNeasy Mini spin column in a new 2 ml collection tube (provided), add 300 μl Buffer AW1, and centrifuge for 3 min at 6000 x g (8000 rpm).
9. Discard flow-through and collection tube. Once collection tube changed repeat step 8.
10. Place the DNeasy Mini spin column in a new 2 ml collection tube (provided), add 300 μl Buffer AW2, and centrifuge for 5 min at 20,000 x g (14,000 rpm (full-speed)) to dry the DNeasy membrane. Discard flow-through and collection tube. Once collection tube changed repeat step 10. It is important to dry the membrane of the DNeasy Mini spin column, since residual ethanol may interfere with subsequent reactions. This centrifugation step ensures that no residual ethanol will be carried over during the following elution.
11. Following the centrifugation step, remove the DNeasy Mini spin column carefully so that the column does not come into contact with the flow-through, since this will result in carryover of ethanol. If carryover of ethanol occurs, empty the collection tube, then reuse it in another centrifugation for 1 min at 20,000 x g (14,000 rpm).
12. Place the DNeasy Mini spin column in a clean 1.5 ml or 2 ml micro centrifuge tube then centrifuge at max speed for 5 mins. Buffer AE should be warmed.
13. Pipette 50 μl Buffer AE directly onto the DNeasy membrane. Incubate at room temperature for 15 min, and then centrifuge for 3min at 6000 x g (8000 rpm) to elute. Repeat step 13 with a new clean 1.5 ml or 2 ml micro centrifuge tube and store both samples.

**DNA extraction protocol from equine hair roots bulbs using Qiagen Gentra Puregene reagents**

***Reagents***

- 1.5ml centrifuge tubes
- Cell lysis solution (Qiagen)
- Proteinase K
- Protein precipitation solution (PPS) (Qiagen)
- Isopropanol
- 70% ethanol (EtOH)
- Hydration solution (1x TE)
- Heat block at 57°C

***Method***

1. Select approx. 15 hairs with good roots/bulbs
2. Cut the hair root directly into an centrifuge tube and label correctly
3. Add 300µl cell lysis solution and 5µl proteinase K (can make up a master mix if have multiple samples)
4. Vortex for approx. 45 seconds, brief centrifuge to ensure the hair is all submerged
5. Leave overnight on the heat block
6. Collect ice
7. Transfer the supernatant to a new centrifuge tube and label
8. Add 100µl PPS and vortex for 45 seconds, quick spin and put on ice for 6-10 minutes
9. Centrifuge for 3.5 mins at 16,000g
10. Add 300µl isopropanol to fresh centrifuge tube and label
11. Pour supernatant into isopropanol tube
12. Gently invert approx. 40 times and centrifuge again for 3.5 mins at 16,000g
13. Discard supernatant and add 300µl EtOH
14. Vortex for approx. 45 seconds and centrifuge for 3.5 mins at 16,000g
15. Discard EtOH and dab the top of the tube on a paper towel
16. Allow EtOH to evaporate.
17. Once dry, add hydration solution (40µl for large pellet, 25µl for smaller pellet)
18. Leave at RT for 1-2 hours, vortexing for 5 secs every 30 mins
19. Quantify using Nanodrop

**DNA extraction protocol from equine whole blood using the Illustra Nucleon BACC kit**

***Reagents***

- Reagent A (Illustra) (supplied as a 4x concentrate. Prior to first use it should be diluted four fold with deionized water and autoclaved, at 121˚C 15 psi for 15 minutes, in suitable aliquots.)
- Reagent B (Illustra)
- Nucleon resin (Illustra)
- 5M Sodium perchlorate (Illustra)
- 1.5 mL centrifuge tubes
- Heat block at
- Chloroform (analytical grade)
- 70% and absolute ethanol
- Xylene
- Dry ice
- TE buffer

***Method***

1. Collect the blood in sodium EDTA tubes.
2. Using an aseptic procedure add 4 times the volume of Reagent A to the blood sample. Rotary mix for 4 minutes at room temperature. Centrifuge at 1300 g for 5 minutes. Discard the supernatant.
3. To the pellet add 2 mL of Reagent B. Vortex briefly to resuspend the pellet. Transfer the suspension to a 15 mL centrifuge tube.
4. Add 500 μL of sodium perchlorate solution. Mix by hand, inverting the capped tube at least 7 times.
5. Add 2 mL of chloroform. Mix by hand, inverting the capped tube at least 7 times.
6. Holding the tube vertically without disturbing the Nucleon resin layer (brown in color), transfer the upper phase (approximately 2.5 mL) to a clean tube of minimum volume 7.5 mL.
7. Add 2 volumes of cold absolute ethanol. Mix by inversion until the precipitate appears.
8. Centrifuge at top speed (minimum 4000 g) for 5 minutes to pellet the DNA. Discard the supernatant.
9. Add 2 mL cold 70% (v/v) ethanol, mix several times by inversion. Re-centrifuge and discard the supernatant. This step can be repeated if necessary.
10. Air dry the pellet for 10 minutes, ensuring that all theethanol has been removed. Re-dissolve the DNA in an appropriate volume of water or TE buffer (e.g. 1.0–2.0 mL). The DNA should re-dissolve within 2 hours when using a rotary mixer.
