# Supplementary material for: Genetic characterisation of the Connemara pony and the Warmblood horse using a within-breed clustering approach
Source: Genet Sel Evol. 2023 Aug 17;55:60. doi: 10.1186/s12711-023-00827-w (PMC10436415; doi:10.1186/s12711-023-00827-w)
Supplement: Supplementary file 2 — Additional file 2:Table S1. Horse breed and sample type. Table indicating the breed, breed subtype, sample origin, sample type and genotyping platform of each sample. CP: Connemara pony; WB: Warmblood horse; KWPN: Koninklijk Warmbloed Paardenstamboek Nederland; M: male; F: female; WGS: whole genome sequencing; SNP: single nucleotide polymorphism array genotyping panel. [file 12711_2023_827_MOESM2_ESM.docx]

| **Additional file 2: Table S1: Horse breed and sample type** | | | | | | | |
| --- | --- | --- | --- | --- | --- | --- | --- |
| **Sample ID** | **Breed** | **Breed subtype** | **Age** | **Sex** | **Sample origin** | **Sample type** | **Platform** |
| 002 | CP |  | 10 | M | UK | Hair root | WGS |
| 003 | WB | Trakehner x WB | 19 | F | UK | Hair root | WGS |
| 004 | WB | Trakehner | 6 | M | UK | Muscle | WGS |
| 005 | WB |  | 12 | M | UK | Muscle | SNP |
| 006 | WB | KWPN | 9 | M | UK | Hair root | SNP |
| 007 | CP |  | 17 | M | UK | Muscle | SNP |
| 008 | CP |  | 7 | F | UK | Hair root | WGS +SNP |
| 009 | WB | British WB | 13 | M | UK | Muscle | SNP |
| 010 | WB | KWPN | 9 | M | UK | Whole blood | SNP |
| 012 | WB | Holsteiner | 8 | M | UK | Hair root | WGS |
| 013 | WB | Hanoverian | 14 | M | UK | Hair root | WGS |
| 014 | WB |  | 6 | F | UK | Muscle | WGS |
| 016 | WB |  | 14 | F | UK | Muscle | SNP |
| 018 | CP |  | 8 | M | UK | Muscle | WGS |
| 020 | WB |  | 6 | M | UK | Muscle | SNP |
| 021 | WB | KWPN | 10 | F | UK | Whole blood | SNP |
| 022 | WB | Holsteiner |  | F | UK | Muscle | SNP |
| 023 | WB | KWPN | 7 | M | UK | Muscle | SNP |
| 024 | WB |  | 11 | F | UK | Muscle | SNP |
| 025 | CP |  | 6 | F | UK | Muscle | WGS |
| 026 | WB |  | 19 | F | UK | Whole blood | WGS |
| 027 | WB | Oldenburg | 11 | M | UK | Muscle | SNP |
| 031 | CP |  | 11 | F | UK | Hair root | WGS |
| 032 | CP |  | 8 | M | UK | Muscle | WGS |
| 033 | WB | British WB | 10 | F | UK | Hair root | SNP |
| 034 | CP |  | 9 | M | UK | Hair root | WGS |
| 035 | WB | KWPN | 8 | F | UK | Hair root | SNP |
| 037 | CP |  | 13 | F | UK | Muscle | SNP |
| 038 | WB |  | 7 | M | UK | Muscle | SNP |
| 040 | WB | Zangersheide | 11 | F | UK | Hair root | SNP |
| 041 | CP |  | 11 | M | UK | Muscle | WGS |
| 042 | WB | KWPN | 10 | F | UK | Muscle | SNP |
| 043 | WB |  | 8 | M | UK | Muscle | SNP |
| 044 | WB | KWPN | 15 | M | UK | Hair root | SNP |
| 045 | WB |  | 16 | F | UK | Hair root | WGS |
| 046 | WB | Belgian WB | 15 | M | UK | Hair root | SNP |
| 047 | WB | Hanoverian | 8 | F | UK | Muscle | WGS +SNP |
| 048 | WB |  | 6 | F | UK | Muscle | SNP |
| 050 | CP |  | 8 | M | UK | Muscle | WGS +SNP |
| 051 | CP |  | 8 | M | UK | Hair root | WGS |
| 052 | CP |  | 17 | M | UK | Hair root | WGS |
| 053 | CP |  | 12 | M | UK | Hair root | SNP |
| 054 | WB | Selle Francais | 13 | M | UK | Muscle | WGS |
| 055 | WB |  | 10 | F | UK | Muscle | SNP |
| 056 | WB | Belgian WB | 12 | M | UK | Hair root | SNP |
| 057 | CP |  | 20 | M | UK | Hair root | WGS |
| 058 | WB | KWPN | 8 | M | UK | Hair root | SNP |
| 060 | WB | KWPN | 8 | M | UK | Muscle | WGS |
| 061 | CP |  |  | F | UK | Muscle | SNP |
| 062 | WB |  | 5 | F | UK | Muscle | WGS |
| 063 | WB | Dutch WB X | 7 | M | UK | Muscle | SNP |
| 064 | CP |  | 10 | F | UK | Muscle | SNP |
| 065 | WB | KWPN x Selle Francais | 10 | M | UK | Hair root | SNP |
| 066 | WB | Trakehner | 6 | F | UK | Muscle | SNP |
| 067 | CP |  | 6 | M | UK | Muscle | SNP |
| 068 | WB |  | 12 | M | UK | Muscle | SNP |
| 069 | WB |  | 4 | F | UK | Muscle | SNP |
| 070 | WB | Selle Francais | 8 | F | UK | Muscle | SNP |
| 071 | WB | Anglo European | 12 | M | UK | Hair root | SNP |
| 072 | CP |  | 5 | M | UK | Muscle | WGS |
| 074 | WB | Belgian WB | 20 | F | UK | Hair root | WGS |
| 075 | WB |  | 12 | F | UK | Muscle | WGS |
| 076 | WB | Oldenburg | 8 | M | UK | Hair root | SNP |
| 077 | CP |  | 11 | F | UK | Muscle | WGS |
| 078 | WB |  | 6 | M | UK | Muscle | SNP |
| 079 | WB |  | 10 | F | UK | Muscle | WGS |
| 080 | WB | Polish WB X | 18 | M | UK | Hair root | WGS |
| 081 | WB | KWPN | 8 | M | UK | Hair root | SNP |
| 082 | WB | Anglo European | 8 | M | UK | Hair root | SNP |
| 083 | WB | Anglo European | 9 | M | UK | Hair root | SNP |
| 084 | CP |  | 8 | M | UK | Whole blood | SNP |
| 085 | CP |  | 26 | F | UK | Whole blood | SNP |
| 086 | WB | Belgian WB | 9 | M | UK | Muscle | SNP |
| 087 | CP |  | 19 | M | UK | Hair root | SNP |
| 089 | WB |  |  | F | UK | Muscle | SNP |
| 091 | CP |  | 11 | M | UK | Hair root | SNP |
| 092 | WB |  | 5 | F | UK | Muscle | SNP |
| 093 | WB | KWPN | 12 | F | UK | Hair root | SNP |
| 094 | WB | Trakehner | 16 | M | UK | Whole blood | SNP |
| 095 | WB |  | 9 | M | UK | Muscle | SNP |
| 096 | WB | KWPN | 7 | M | UK | Hair root | WGS |
| 098 | WB | Zangersheide | 8 | M | UK | Hair root | SNP |
| 099 | CP |  | 10 | F | UK | Muscle | WGS |
| 100 | CP |  | 9 | M | UK | Muscle | WGS |
| 101 | WB | KWPN | 17 | F | UK | Hair root | SNP |
| 103 | WB | Anglo European | 11 | M | UK | Hair root | SNP |
| 104 | WB | KWPN | 11 | F | UK | Hair root | SNP |
| 106 | WB | Slovakian WB | 8 | M | UK | Hair root | WGS |
| 107 | WB | Anglo European | 8 | M | UK | Hair root | SNP |
| 108 | WB | Anglo European | 21 | M | UK | Hair root | SNP |
| 111 | WB | Zangersheide | 19 | M | UK | Hair root | SNP |
| 113 | WB | British WB | 10 | F | UK | Hair root | SNP |
| 114 | WB | British WB | 14 | F | UK | Hair root | SNP |
| 115 | WB | Oldenburg | 8 | M | UK | Muscle | SNP |
| 116 | WB | KWPN | 17 | M | UK | Hair root | SNP |
| 117 | CP |  | 9 | M | UK | Hair root | SNP |
| 118 | WB |  | 8 | M | UK | Muscle | SNP |
| 119 | WB | Anglo European | 9 | F | UK | Hair root | SNP |
| 120 | WB |  |  | M | UK | Hair root | SNP |
| 121 | WB | Anglo European | 9 | F | UK | Hair root | SNP |
| 122 | CP |  | 21 | F | UK | Hair root | WGS |
| 123 | WB | Hanoverian | 8 | M | UK | Hair root | SNP |
| 124 | CP |  | 14 | M | UK | Hair root | SNP |
| 125 | WB |  | 9 | F | UK | Muscle | SNP |
| 126 | WB | Anglo European | 8 | F | UK | Hair root | SNP |
| 127 | CP |  | 15 | F | UK | Hair root | SNP |
| 128 | WB | Westphalian | 11 | F | UK | Hair root | SNP |
| 129 | CP |  | 17 | F | UK | Hair root | WGS |
| 130 | WB |  |  | F | UK | Hair root | SNP |
| 131 | WB | PSI | 2 | F | UK | Muscle | SNP |
| 133 | WB |  |  | F | UK | Muscle | SNP |
| 134 | WB | Belgian WB | 7 | M | UK | Hair root | SNP |
| 135 | WB | Belgian WB | 17 | M | UK | Hair root | SNP |
| 136 | WB |  | 7 | F | UK | Muscle | SNP |
| 137 | WB | KWPN | 13 | M | UK | Hair root | SNP |
| 138 | CP |  | 8 | M | UK | Hair root | WGS |
| 139 | WB | Selle Francais | 17 | M | UK | Hair root | SNP |
| 150 | WB |  | 14 | F | UK | Muscle | SNP |
| 151 | CP |  | 10 | M | UK | Muscle | SNP |
| 152 | WB |  | 11 | F | UK | Muscle | SNP |
| 153 | WB |  | 6 | M | UK | Muscle | SNP |
| 154 | WB |  | 7 | M | UK | Muscle | SNP |
| 156 | WB | KWPN | 6 | M | UK | Muscle | SNP |
| 157 | WB | Hanoverian | 8 | M | UK | Muscle | SNP |
| 158 | WB |  | 9 | M | UK | Muscle | SNP |
| 159 | WB |  |  | F | UK | Muscle | SNP |
| 160 | WB |  | 5 | M | UK | Muscle | SNP |
| AV158 | WB | Polish WB |  | M | Continental Europe | Publicly available WGS (Project: PRJEB14779; Sample: ERS1982317) | WGS |
| BW01 | WB | Württemberger |  | M | Continental Europe | Publicly available WGS (Project: PRJEB14779; Sample: ERS1263370) | WGS |
| BY01 | WB | Bavarian |  | M | Continental Europe | Publicly available WGS (Project: PRJEB14779; Sample: ERS1263371) | WGS |
| EMO004 | WB | Holsteiner |  | F | Continental Europe | Publicly available WGS (Project: PRJEB14779; Sample: ERS2642729) | WGS |
| EMO081 | WB | Holsteiner |  |  | Continental Europe | Publicly available WGS (Project: PRJEB14779; Sample: ERS2642731) | WGS |
| EMO082 | WB | Holsteiner |  | F | Continental Europe | Publicly available WGS (Project: PRJEB14779; Sample: ERS2642732) | WGS |
| HAN01 | WB | Hanoverian |  | M | Continental Europe | Publicly available WGS (Project: PRJEB14779; Sample: ERS1263372) | WGS |
| HN001 | WB | Hanoverian |  | M | Continental Europe | Publicly available WGS (Project: PRJEB14779; Sample: ERS1982322) | WGS |
| HOL01 | WB | Holsteiner |  | M | Continental Europe | Publicly available WGS (Project: PRJEB14779; Sample: ERS1263373) | WGS |
| HOL02 | WB | Holsteiner |  | M | Continental Europe | Publicly available WGS (Project: PRJEB14779; Sample: ERS1263374) | WGS |
| HS004 | WB | Holsteiner |  | M | Continental Europe | Publicly available WGS (Project: PRJEB14779; Sample: ERS1982320) | WGS |
| KWPN1 | WB | KWPN |  |  | Continental Europe | Publicly available WGS (Project: PRJEB14779; Sample: ERS1246371) | WGS |
| OL008 | WB | Oldenburg |  | M | Continental Europe | Publicly available WGS (Project: PRJEB14779; Sample: ERS1982321) | WGS |
| OLD01 | WB | Oldenburg |  | M | Continental Europe | Publicly available WGS (Project: PRJEB14779; Sample: ERS1263375) | WGS |
| OLD02 | WB | Oldenburg |  | M | Continental Europe | Publicly available WGS (Project: PRJEB14779; Sample: ERS1263376) | WGS |
| P1 | WB | Holsteiner |  | M | Continental Europe | Publicly available WGS (Project: PRJEB14779; Sample: ERS1263381) | WGS |
| RAO441 | WB | Swiss WB |  | M | Continental Europe | Publicly available WGS (Project: PRJEB14779; Sample: ERS1263383) | WGS |
| SRR1769892 | CP |  |  | M | USA | Publicly available WGS (Project: PRJNA273402; Sample: SRS824998) | WGS |
| SRR1769893 | CP |  |  | M | USA | Publicly available WGS (Project: PRJNA273402; Sample: SRS825118) | WGS |
| SRR1769922 | CP |  |  | F | USA | Publicly available WGS (Project: PRJNA273402; Sample: SRS825119) | WGS |
| SW033 | WB | Swiss WB |  | M | Continental Europe | Publicly available WGS (Project: PRJEB14779; Sample: ERS1982329) | WGS |
| TRA01 | WB | Trakehner |  | M | Continental Europe | Publicly available WGS (Project: PRJEB14779; Sample: ERS1263377) | WGS |
| TRH056 | WB | Trakehner |  | F | Continental Europe | Publicly available WGS (Project: PRJEB14779; Sample: ERS2017586) | WGS |
| WF01 | WB | Westphalian |  | M | Continental Europe | Publicly available WGS (Project: PRJEB14779; Sample: ERS1263379) | WGS |
| WF02 | WB | Westphalian |  | M | Continental Europe | Publicly available WGS (Project: PRJEB14779; Sample: ERS1263380) | WGS |
