# Supplementary material for: Genetic characterisation of the Connemara pony and the Warmblood horse using a within-breed clustering approach
Source: Genet Sel Evol. 2023 Aug 17;55:60. doi: 10.1186/s12711-023-00827-w (PMC10436415; doi:10.1186/s12711-023-00827-w)
Supplement: Supplementary file 3 — Additional file 3: Figure S1. Elbow plot using within-sum of squares and silhouette plot for selection of appropriate number of clusters. Elbow plots (top) and silhouette plots (bottom) for selection of appropriate number of clusters (k) for k-means clustering analysis in WB (left) and CP (right). CP: Connemara pony; WB: Warmblood horse; WSS: within sum of squares; k: number of clusters. [file 12711_2023_827_MOESM3_ESM.docx]

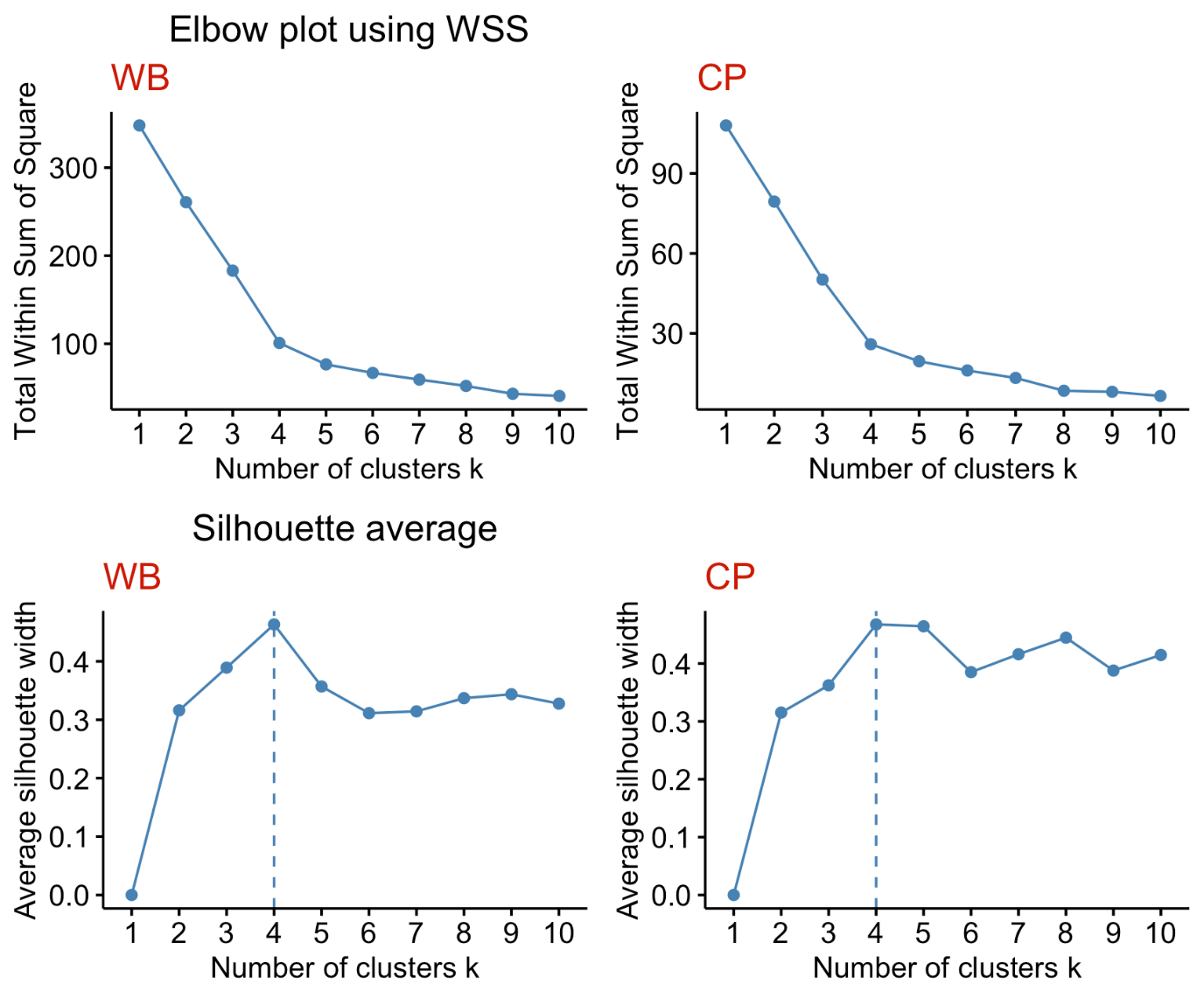


Additional file 3: Figure S1: Elbow plots (top) and silhouette plots (bottom) for selection of appropriate number of clusters (k) for k-means clustering analysis in WB (left) and CP (right).
