# Supplementary material for: Genetic characterisation of the Connemara pony and the Warmblood horse using a within-breed clustering approach
Source: Genet Sel Evol. 2023 Aug 17;55:60. doi: 10.1186/s12711-023-00827-w (PMC10436415; doi:10.1186/s12711-023-00827-w)
Supplement: Supplementary file 4 — Additional file 4: Figure S2. Principal components (PCs) of the genetic relationship matrices for 116 WB (lower diagonal) and 36 CP (upper diagonal). Principal components of the genetic relationship matrices for 116 WB (lower diagonal) and 36 CP (upper diagonal). Upper and lower diagonal plots for CP and WB respectively, with colour designating the k-means assigned cluster and marker designating breed subtype: in CP B) principal component (PC) 1 by PC 2; C) PC 1 by PC 3; and F) PC 2 by PC 3; in WB D) PC 1 by PC 2; G) PC 1 by PC 3; and H) PC 2 by PC 3. Diagonal plots are kernel density estimator plots illustrating the distribution of the principal components, with distribution curves for each cluster: A) PC 1; E) PC 2; and I) PC 3. The first three PC in CP explained 4.7%, 4.2% and 3.7% of variance respectively, and in WB explained 2.5%, 2.2% and 1.7% of variance respectively. CP: Connemara pony; WB: Warmblood horse; UK: United Kingdom; EU: rest of Europe; US: United States; X: unregistered. [file 12711_2023_827_MOESM4_ESM.docx]

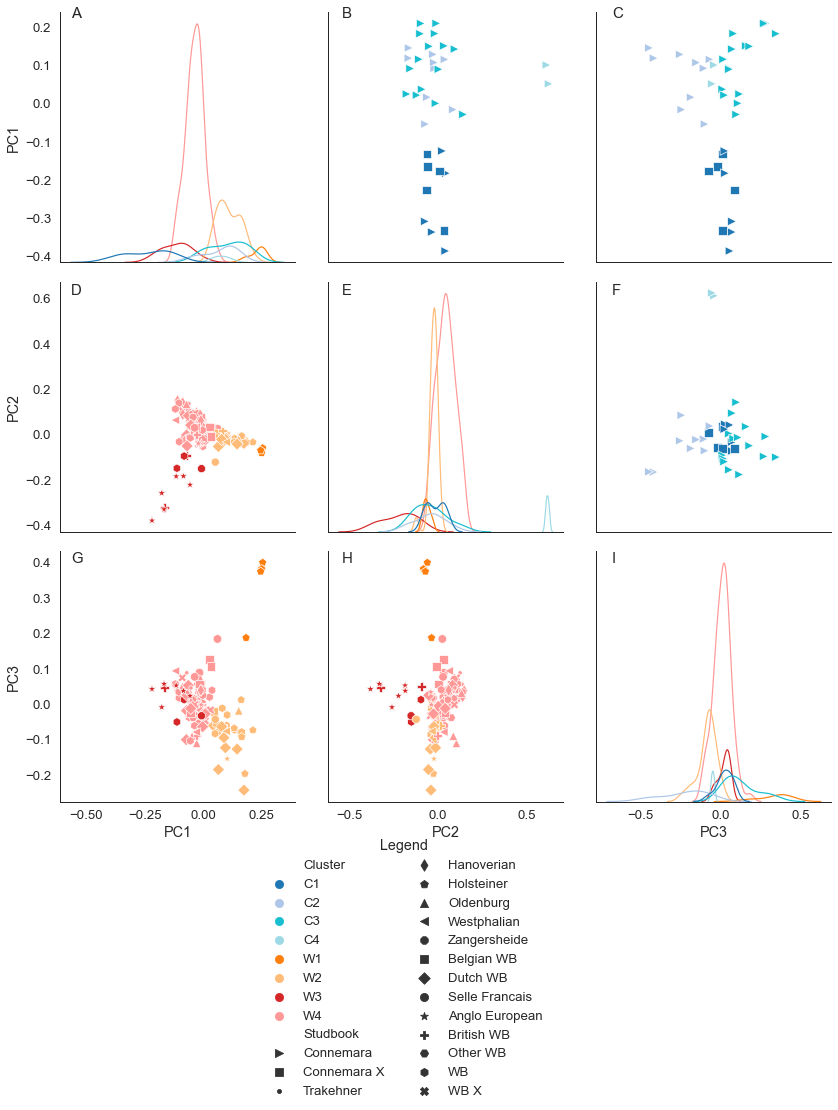


Additional file 4: Figure S2: Principal components of the genetic relationship matrices for 116 WB (lower diagonal) and 36 CP (upper diagonal). Upper and lower diagonal plots for CP and WB respectively, with colour designating the k-means assigned cluster and marker designating breed subtype: in CP B) principal component (PC) 1 by PC 2; C) PC 1 by PC 3; and F) PC 2 by PC 3; in WB D) PC 1 by PC 2; G) PC 1 by PC 3; and H) PC 2 by PC 3. Diagonal plots are kernel density estimator plots illustrating the distribution of the principal components, with distribution curves for each cluster: A) PC 1; E) PC 2; and I) PC 3. The first three PCs in CP explained 4.7%, 4.2% and 3.7% of variance respectively, and in WB explained 2.5%, 2.2% and 1.7% of variance respectively. CP: Connemara pony; WB: Warmblood horse; UK: United Kingdom; EU: rest of Europe; US: United States; X: unregistered.
