# Supplementary material for: Genetic characterisation of the Connemara pony and the Warmblood horse using a within-breed clustering approach
Source: Genet Sel Evol. 2023 Aug 17;55:60. doi: 10.1186/s12711-023-00827-w (PMC10436415; doi:10.1186/s12711-023-00827-w)
Supplement: Supplementary file 5 — Additional file 5: Table S2. Over-representation of breed subtypes and sample origin in CP and WB within-breed genetic groups. Breed subtypes and sample origins significantly associated with different genetic groups using Chi-square testing. CP: Connemara pony; WB: Warmblood horse; UK: United Kingdom; EU: rest of Europe; US: United States; X: unregistered. [file 12711_2023_827_MOESM5_ESM.docx]

| Additional file 5: Table S2: Over-representation of breed subtypes and sample origin in CP and WB within-breed genetic groups | | |
| --- | --- | --- |
| Within-breed genetic group/sample origin group | Number of horses | Overrepresented breed subtypes/sample origin |
| UK CP | 33 |  |
| US CP | 3 |  |
| C1 | 10 | Connemara X |
| C2 | 9 |  |
| C3 | 15 |  |
| C4 | 2 | US CP |
| UK WB | 94 | Anglo European, Dutch WB |
| EU WB | 22 | Holsteiner, Oldenburg, Other WB, Westphalian |
| W1 | 4 | Holsteiner, EU WB |
| W2 | 27 |  |
| W3 | 11 | Anglo European, British WB, UK WB |
| W4 | 74 |  |
