# Supplementary material for: Genetic characterisation of the Connemara pony and the Warmblood horse using a within-breed clustering approach
Source: Genet Sel Evol. 2023 Aug 17;55:60. doi: 10.1186/s12711-023-00827-w (PMC10436415; doi:10.1186/s12711-023-00827-w)
Supplement: Supplementary file 6 — Additional file 6: Table S3. Significant terms associated with genes within 1 Mb of top 0.5% markers. Ontology terms identified as significantly over-represented in genes within 1 Mb of the top 0.5% SNPs between different groups using DAVID. CP: Connemara pony; WB: Warmblood horse; FST: Wright’s fixation index. [file 12711_2023_827_MOESM6_ESM.docx]

| **Additional file 6: Table S3: Significant terms associated with genes within 1 Mb of top 0.5% F_ST_ markers** | | | | |
| --- | --- | --- | --- | --- |
| **Comparison** | **Term type** | **Term** | **Number of involved genes** | **Benjamini-adjusted p-value** |
| Between CP and WB | GO term (CC) | Nucleosome | 41 | 2.2E-4 |
|  | INTERPRO | Histone-fold | 41 | 1.1E-3 |
|  | UP keyword (CC) | Nucleosome core | 19 | 2E-3 |
|  | INTERPRO | Histone core | 29 | 2.1E-3 |
|  | UP keyword (Seq feature) | DOMAIN: Histone | 27 | 4.5E-3 |
|  | KEGG Pathway | Systemic lupus erythematosus | 51 | 2.1E-2 |
|  | UP (Seq feature) | REGION: Disordered | 2787 | 2.2E-2 |
|  | KEGG Pathway | Inflammatory mediator regulation of TRP cells | 39 | 3.8E-2 |
| Between CP genetic groups | INTERPRO | Immunoglobulin V-set | 107 | 6.1E-17 |
|  | SMART | IGv | 67 | 5.2E-10 |
|  | INTERPRO | Immunoglobulin-like fold | 163 | 1.9E-9 |
|  | GO Term (BP) | cell surface receptor signaling pathway | 40 | 1.6E-8 |
|  | UP keyword (seq feature) | DOMAIN:Ig-like | 120 | 1.2E-7 |
|  | INTERPRO | Immunoglobulin-like domain | 120 | 1.3E-7 |
|  | UP keyword (BP) | Olfaction | 116 | 2.7E-7 |
|  | UP keyword (BP) | Sensory transduction | 117 | 1.2E-6 |
|  | GO term (CC) | plasma membrane | 327 | 2.7E-6 |
|  | GO term (MF) | olfactory receptor activity | 132 | 6.2E-6 |
|  | GO term (MF) | G-protein coupled receptor activity | 151 | 2.5E-5 |
|  | INTERPRO | Olfactory receptor | 131 | 4.8E-5 |
|  | SMART | IFabd | 12 | 1.4E-4 |
|  | GO term (BP) | T cell activation involved in immune response | 12 | 1.4E-4 |
|  | GO term (MF) | type I interferon receptor binding | 11 | 1.4E-4 |
|  | UP keyword (CC) | Cell membrane | 165 | 1.5E-4 |
|  | INTERPRO | Interferon alpha/beta/delta | 12 | 3.5E-4 |
|  | GO term (BP) | natural killer cell activation involved in immune response | 11 | 3.5E-4 |
|  | UP keyword (Seq feature) | DOMAIN: IGv | 17 | 7.7E-4 |
|  | GO term (BP) | positive regulation of peptidyl-serine phosphorylation of STAT protein | 11 | 8.8E-4 |
|  | INTERPRO | Homeodomain, metazoa | 23 | 1.3E-3 |
|  | GO term (BP) | B cell differentiation | 17 | 1.4E-3 |
|  | GO term (BP) | B cell proliferation | 12 | 3.9E-3 |
|  | GO term (BP) | humoral immune response | 13 | 4E-3 |
|  | GO term (BP) | response to exogenous dsRNA | 12 | 5.9E-3 |
|  | INTERPRO | G protein-coupled receptor, rhodopsin-like | 145 | 8.7E-3 |
|  | SMART | HOX | 36 | 1.3E-2 |
|  | KEGG Pathway | Human papillomavirus infection | 47 | 2.6E-2 |
|  | INTERPRO | Homeodomain | 37 | 2.6E-2 |
|  | UP keyword (Seq feature) | DOMAIN: G Protein receptor F1 2 | 146 | 3E-2 |
|  | UP keyword (Seq feature) | DOMAIN: Homeobox | 36 | 3E-2 |
|  | UP keyword (Seq feature) | DNA_BIND: Homeobox | 36 | 3E-2 |
|  | INTERPRO | GPCR, rhodopsin-like, 7TM | 146 | 3.4E-2 |
| Between WB genetic groups | INTERPRO | Immunoglobulin V-set | 184 | 4.5E-5 |
|  | SMART | IGv | 123 | 2.5E-3 |
|  | UP keyword (Seq feature) | Domain: IGv | 28 | 3.2E-2 |
| Between C1 and other CP groups | INTERPRO | Immunoglobulin V-set | 193 | 6.1E-7 |
|  | SMART | IGv | 121 | 5.4E-3 |
|  | INTERPRO | High sulphur keratin-associated protein | 20 | 2.7E-2 |
| Between C2 and other CP groups | INTERPRO | Immunoglobulin V-set | 165 | 2.3E-20 |
|  | UP keyword (Seq feature) | DOMAIN:Ig-like | 209 | 9.7E-13 |
|  | INTERPRO | Immunoglobulin-like domain | 209 | 1.1E-12 |
|  | INTERPRO | Immunoglobulin-like fold | 273 | 1.1E-12 |
|  | SMART | IGv | 109 | 1.1E-12 |
|  | GO term (BP) | cell surface receptor signaling pathway | 56 | 8.4E-8 |
|  | INTERPRO | Immunoglobulin subtype | 149 | 7.9E-7 |
|  | SMART | IG | 149 | 2E-6 |
|  | SMART | SM01394 | 15 | 2.1E-3 |
|  | INTERPRO | S100/CaBP-9k-type, calcium binding, subdomain | 15 | 4.6E-3 |
|  | GO term (BP) | response to bacterium | 165 | 1.1E-2 |
| Between C3 and other CP groups | SMART | IGv | 105 | 3.3E-2 |
|  | INTERPRO | Immunoglobulin V-set | 152 | 3.7E-2 |
| Between W2 and other WB groups | INTERPRO | Immunoglobulin V-set | 125 | 1.1E-3 |
|  | GO term (BP) | cellular response to interferon-gamma | 26 | 7.9E-3 |
|  | GO term (BP) | response to bacterium | 35 | 8.3E-3 |
|  | SMART | IGv | 84 | 1.7E-2 |
|  | GO term (BP) | lymphocyte chemotaxis | 15 | 3.2E-2 |
|  | GO term (BP) | cellular response to interleukin-1 | 22 | 3.2E-2 |
|  | INTERPRO | Beta defensin type | 10 | 4.1E-2 |
|  | GO term (MF) | CCR chemokine receptor binding | 14 | 4.8E-2 |
| Between W3 and other WB groups | UP keyword (Seq feature) | COMPBIA: Polar residues | 121 | 5.4E-6 |
|  | UP keyword (Seq feature) | REGION: Disordered | 195 | 9.2E-4 |
|  | UP keyword (Seq feature) | COMPBIAS: Basic and acidic residues | 96 | 4.3E-3 |
| Between W4 and other WB groups | INTERPRO | Immunoglobulin V-set | 166 | 2.1E-5 |
|  | SMART | IGv | 116 | 6.4E-5 |
|  | SMART | SM01394 | 19 | 3.3E-4 |
|  | GO term (BP) | response to bacterium | 45 | 9.9E-4 |
|  | INTERPRO | S100/CaBP-9k-type, calcium binding, subdomain | 19 | 1.3E-3 |
|  | INTERPRO | High sulphur keratin-associated protein | 20 | 1.4E-3 |
|  | UP keyword (CC) | Nucleus | 399 | 2.9E-2 |
|  | PIR superfamily | transmembrane serine protease, TMPRSS11A type | 8 | 2.9E-2 |
|  | SMART | IG | 176 | 3.2E-2 |
|  | INTERPRO | Immunoglobulin subtype | 176 | 3.4E-2 |
| GO: Gene Ontology term (55, 59); KEGG: Kyoto Encyclopaedia of Genes and Genomes pathway term (56-58) | | | | |
