# Supplementary material for: Genetic characterisation of the Connemara pony and the Warmblood horse using a within-breed clustering approach
Source: Genet Sel Evol. 2023 Aug 17;55:60. doi: 10.1186/s12711-023-00827-w (PMC10436415; doi:10.1186/s12711-023-00827-w)
Supplement: Supplementary file 7 — Additional file 7: Table S4. Largest number of individuals and % of the group sharing a run of homozygosity (ROH) in CP and WB, and in within-breed genetic groups. CP: Connemara pony; WB: Warmblood horse; ROH: runs of homozygosity. [file 12711_2023_827_MOESM7_ESM.docx]

| **Additional file 7: Table S4: Largest number of individuals and % of the group sharing a run of homozygosity (ROH) in CP and WB, and in within-breed genetic groups** | | |
| --- | --- | --- |
| **Group** | **Highest % of individuals sharing a ROH** | **Largest number of individuals sharing a ROH** |
| All CP | 22.2% | 8 |
| All WB | 18.1% | 21 |
| UK CP | 24.2% | 8 |
| C1 | 30% | 3 |
| C2 | 44.4% | 4 |
| C3 | 20% | 3 |
| C4 | 50% | 1 |
| UK WB | 17.0% | 16 |
| EU WB | 27.3% | 6 |
| W1 | 50% | 2 |
| W2 | 25.9% | 7 |
| W3 | 27.3% | 3 |
| W4 | 23.0% | 17 |
