# Supplementary material for: Genetic characterisation of the Connemara pony and the Warmblood horse using a within-breed clustering approach
Source: Genet Sel Evol. 2023 Aug 17;55:60. doi: 10.1186/s12711-023-00827-w (PMC10436415; doi:10.1186/s12711-023-00827-w)
Supplement: Supplementary file 8 — Additional file 8: Table S5. Significant ontology terms associated with genes within ROH regions present in > 10% of horses belonging to a given genetic group, origin group or breed. Ontology terms identified as significantly over-represented in genes within 1 Mb of the ROH regions present in > 10% of horses belonging to a given genetic group, origin group or breed using DAVID. CP: Connemara pony; WB: Warmblood horse; ROH: runs of homozygosity. [file 12711_2023_827_MOESM8_ESM.docx]

| **Additional file 8: Table S5: Significant ontology terms associated with genes within ROH regions present in ≥10% of horses belonging to a given genetic group, origin group or breed** | | | | |
| --- | --- | --- | --- | --- |
| **Gene set** | **Term type** | **Term** | **Number of genes** | **Benjamini-adjusted p-value** |
| CP-unique | - | - | - | - |
| WB-unique | - | - | - | ^-^ |
| Shared between CP and WB | - | - | - | ^-^ |
| UK CP | KEGG Pathway | Nitrogen metabolism | 5 | 2.7E-2 |
| C1 | - | - | - | - |
| C2 | INTERPRO | Intercellular adhesion molecule, N-terminal | 4 | 2.2E-3 |
|  | INTERPRO | Intercellular adhesion molecule/vascular cell adhesion molecule, N-terminal | 4 | 2.2E-3 |
|  | INTERPRO | Intercellular adhesion molecule | 3 | 2.5E-2 |
| C3 | - | - | - | - |
| European WB | INTERPRO | Type II keratin | 13 | 1.8E-7 |
|  | GO Term | Keratin filament | 13 | 2.8E-5 |
|  | UP Keyword | Intermediate filament | 15 | 2.5E-4 |
|  | SMART | SM01391 | 15 | 8.0E-4 |
|  | INTERPRO | Intermediate filament, conserved site | 14 | 1.1E-3 |
|  | INTERPRO | Intermediate filament protein | 15 | 1.1E-3 |
| UK WB | - | - | - | - |
| W1 | INTERPRO | Neurotransmitter-gated ion channel transmembrane domain | 5 | 2.6E-3 |
|  | INTERPRO | Neurotransmitter-gated ion channel conserved site | 5 | 2.6E-3 |
|  | INTERPRO | Neurotransmitter-gated ion channel | 5 | 2.6E-3 |
|  | INTERPRO | Neurotransmitter-gated ion channel ligand binding | 5 | 2.6E-3 |
|  | KEGG Pathway | Neurotransmitter-gated ion channel interaction | 8 | 8.3E-3 |
|  | UP Keyword | Synapse | 5 | 9.8E-3 |
|  | UP Keyword | Ion transport | 7 | 1.9E-2 |
|  | UP Keyword | Ion channel | 6 | 1.9E-2 |
|  | UP Keyword | Cell junction | 5 | 1.9E-2 |
|  | GO Term | Cell junction | 6 | 2.1E-2 |
|  | UP Keyword | Lipid binding | 3 | 3.2E-2 |
| W2 | UP Keyword | FAD | 6 | 4.9E-2 |
|  | UP Keyword | Flavoprotein | 6 | 4.9E-2 |
| W3 | - | - | - | - |
| W4 | - | - | - | - |
| GO: Gene Ontology term (55, 59); INTERPRO: EMBL-EBI InterPro database term (68); KEGG: Kyoto Encyclopaedia of Genes and Genomes pathway term (56-58); UP: UniProt keywords (69) | | | | |
