# Supplementary material for: Genetic characterisation of the Connemara pony and the Warmblood horse using a within-breed clustering approach
Source: Genet Sel Evol. 2023 Aug 17;55:60. doi: 10.1186/s12711-023-00827-w (PMC10436415; doi:10.1186/s12711-023-00827-w)
Supplement: Supplementary file 9 — Additional file 9: Figure S3. Mean genomic inbreeding (as FROH) by chromosome, illustrated in within-breed genetic group. Mean genomic inbreeding (as FROH) by chromosome, illustrated in within-breed genetic group. CP: Connemara pony; WB: Warmblood horse; ROH: runs of homozygosity. [file 12711_2023_827_MOESM9_ESM.docx]

Additional file 9: Figure S3: Mean genomic inbreeding (as F_ROH_) by chromosome, illustrated within breed genetic group.
